# Design, Synthesis, In Silico Docking, Multitarget Bioevaluation and Molecular Dynamic Simulation of Novel Pyrazolo[3,4-*d*]Pyrimidinone Derivatives as Potential In Vitro and In Vivo Anti-Inflammatory Agents

**DOI:** 10.3390/ph18091326

**Published:** 2025-09-04

**Authors:** Mostafa Roshdi, Mamdouh F. A. Mohamed, Eman A. M. Beshr, Hossameldin A. Aziz, Sahar M. Gebril, Stefan Bräse, Aliaa M. Mohassab

**Affiliations:** 1 Department of Medicinal Chemistry, Faculty of Pharmacy, Minia University, Minia 61519, Egypt; mostafa.roshdi1997@gmail.com (M.R.); emanbeshr5@gmail.com (E.A.M.B.); alyaa.mohasab@mu.edu.eg (A.M.M.); 2 Department of Medicinal Chemistry, Faculty of Pharmacy, Merit University, Sohag 82755, Egypt; 3 Department of Pharmaceutical Chemistry, Faculty of Pharmacy, Sohag University, Sohag 82524, Egypt; 4 Department of Pharmaceutical Chemistry, Faculty of Pharmacy, New Valley University, Al-Kharga 72511, Egypt; hossamaziz85@pha.nvu.edu.eg; 5 Medicinal Chemistry Department, Faculty of Pharmacy, Minia National University, New Minia 61768, Egypt; 6 Department of Histology and Cell Biology, Faculty of Medicine, Sohag University, Sohag 82524, Egypt; sahermohamed@med.sohag.edu.eg; 7 Institute for Biological and Chemical System, Karlsruhe Institute of Technology, 76131 Karlsruhe, Germany

**Keywords:** pyrazolo[3,4-*d*]pyrimidine, anti-inflammatory, COX-1, COX-2, TNF-α, IL-6

## Abstract

**Background:** A novel series of pyrazolo[3,4-*d*]pyrimidinone derivatives were synthesized, characterized, and examined for their anti-inflammatory effects. **Results:** The findings indicated that compounds **5d**, **5j**, **5k**, and **5m** demonstrated significant anti-inflammatory effects through the selective inhibition of the COX-2 isozyme, with IC_50_ values ranging from 0.27 to 2.34 μM, compared to celecoxib (IC_50_ = 0.29 μM). Compound **5k** emerged as the most potent, exhibiting a selectivity index (SI) of 95.8 for COX-2 relative to COX-1. In vivo tests additionally validated that compounds **5j** and **5k** demonstrated significant anti-inflammatory efficacy, exhibiting greater suppression percentages of generated paw edema than indomethacin, comparable to celecoxib, while preserving excellent safety profiles with intact gastric tissue. Mechanistic studies demonstrated that the anti-inflammatory efficacy of the target compounds was associated with a substantial decrease in serum levels of TNF-α and IL-6. Moreover, molecular modeling investigations corroborated the in vitro findings. Compound **5k** displayed a binding free energy ΔG of −10.57 kcal/mol, comparable to that of celecoxib, which showed a ΔG of −10.19 kcal/mol. The intensified binding contacts in the COX-2 isozyme indicated the augmented inhibitory efficacy of **5k**. **Conclusions:** Compound **5k** exhibited dual activity by inhibiting the COX-2 isozyme and suppressing the pro-inflammatory cytokines TNF-α and IL-6, therefore providing a remarkable anti-inflammatory effect with increased therapeutic potential.

## 1. Introduction

Inflammation is the body’s multifaceted biological response to protect itself from detrimental stimuli, including infections, damaged cells, or irritants. Acute inflammation is essential to the immune system’s defense and aids in healing; however, chronic or uncontrolled inflammation is associated with several disorders, including cancer, Alzheimer’s disease, rheumatoid arthritis, type II diabetes, and asthma [1,2,3,4]. Despite the complexities of comprehending the signaling pathways involved in the inflammatory response, it remains a compelling challenge to discover effective therapeutic approaches that mitigate its detrimental side effects [5,6]. Cellular and vascular cascades, the fundamental components of the inflammatory process, are initiated by chemical substances known as inflammatory mediators, including cytokines, chemokines, eicosanoids, and products of the proteolytic cascade [7,8]. The dysregulation of pro-inflammatory cytokines, such as tumor necrosis factor-alpha (TNF-α) and interleukins (ILs), has been linked to various diseases, including inflammation, cancer, and autoimmune disorders [9,10,11]. Additionally, the use of broad-spectrum anti-inflammatory and antioxidant therapy for diseases triggered by inflammatory storms by programmed self-derived cryo-dead neutrophils has been documented [12].

Cyclooxygenase (COX) enzyme, also called prostaglandin endoperoxide H synthase (PGHS), plays a pivotal role in inflammation via catalyzing the oxidative conversion of arachidonic acid (AA) into prostaglandin H_2_ (PGH_2_), which is then converted into a wide variety of products, such as thromboxane A_2_ (TxA_2_) and prostaglandins E_2_ (PGE_2_), which have a key role in the inflammatory response [13,14]. Furthermore, the cyclooxygenase (COX) enzyme in humans exists in two distinct isozymes: constitutive COX-1, which is responsible for the protection of gastric mucosa, platelet aggregation, and renal homeostasis, and inducible COX-2, a key inflammatory mediator, which is mainly associated with the production of prostaglandins (prostaglandin E2 and prostaglandin B), thromboxane, and prostacyclin, and pro-inflammatory cytokines including TNF-α and interleukins (ILs), including IL-6 and IL-10, which are implicated in the induction of inflammation [15,16,17]. Therefore, the distinct roles of COX-1 and COX-2 isozymes provide a sought-after therapeutic target to alleviate inflammation and its repercussions while circumventing the undesirable side effects linked to COX-1 inhibition [18,19]. Conventional non-steroidal anti-inflammatory drugs (NSAIDs), such as aspirin, indomethacin, and diclofenac, are the most frequently prescribed medications for treating inflammation, primarily due to their ability to block the arachidonic acid pathway [20,21]. Nonetheless, long-term use of these drugs may cause gastrointestinal toxicity and platelet function disorder arising from their non-selective inhibition of both COX-1 and COX-2 isozymes [22,23]. In addition, selective COX-2 inhibitors, such as COXIBs, were found to possess much better GI tolerability when compared to non-selective COX-1 inhibitors. However, they showed serious cardiovascular (CV) toxicity during their post marketing outcome studies [24]. Moreover, certain COXIBs have variable and challenging pharmacokinetic properties, such as the rate of absorption, extent of plasma protein binding, routes of excretion, and metabolic pathways [25,26]. Consequently, one of the major obstacles confronting medicinal chemists is the development of novel, safe, and effective therapeutic agents for treating inflammation, while simultaneously minimizing the unfavorable GI and CV side effects and implementing adequate pharmacokinetic properties [27,28].

**Figure 1:**
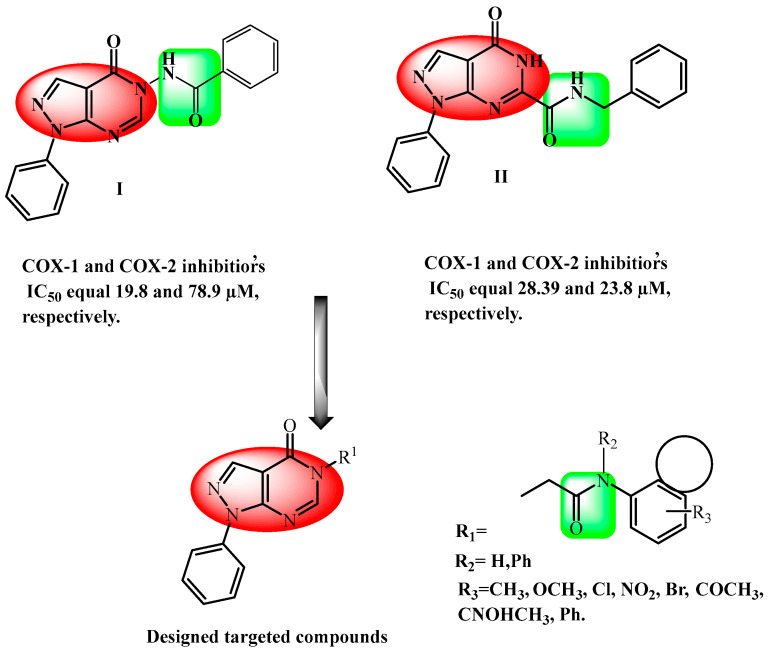
Structures of representative compounds **I–II** and the designed targeted compounds.

The medicinal chemistry community has shown a significant interest in the pyrazolo[3,4-*d*]pyrimidine scaffold as a promising pharmacophore for drug design and discovery. It can mimic the hinge region binding interactions within the kinase active site, as it is considered a bioisostere of the adenine ring of adenosine triphosphate (ATP) [29,30]. This scaffold has exhibited a wide range of pharmacological and biological activities, including antimicrobial, antitubercular, anti-inflammatory, antimalarial, antiviral, and antidiabetic properties [31]. Moreover, research has shown that the pyrazolo[3,4-*d*]pyrimidine nucleus exhibits anti-inflammatory properties [32,33]. Pyrazolo[3,4-*d*]pyrimidine, when fused or linked to other heterocyclic ring systems, serves as an essential scaffold in numerous pharmacologically active compounds [34], including those with analgesic and anti-inflammatory properties, exhibiting significant selectivity for COX-2 over COX-1 [35,36]. Moreover, molecules featuring an acetamide linker or its derivatives as fundamental structures have garnered significant attention owing to their potential therapeutic applications, such as their anti-inflammatory activity [37,38]. For instance, compounds **I** and **II** have emerged as promising anti-inflammatory agents, displaying COX-2 IC_50_ values of 78.9 and 23.8 μM, respectively Figure 1. These values highlight their potency relative to NS398 (IC_50_ = 54.0 μM) and celecoxib (IC_50_ = 54.0 μM) as reference drugs [39].

This study presents the synthesis of novel *N*-5-substituted pyrazolo[3,4-*d*]pyrimidinone derivatives, building on previous findings as part of our ongoing efforts to develop physiologically active heterocyclic molecules. The compounds were examined for their anti-inflammatory properties using in vitro inhibition assays of COX-1 and COX-2, as well as in vivo models, including histopathological analyses. The effects of these substances on the pro-inflammatory cytokines, TNF-α and IL-6, were examined to clarify their mechanism of action. Molecular docking studies elucidated the binding interactions of the most active compounds with COX isoforms. At the same time, computational assessments of their pharmacokinetic profiles and toxicity underscored their drug-likeness and therapeutic promise.

## 2. Results and Discussion

### 2.1. Chemistry

**Scheme 1:**
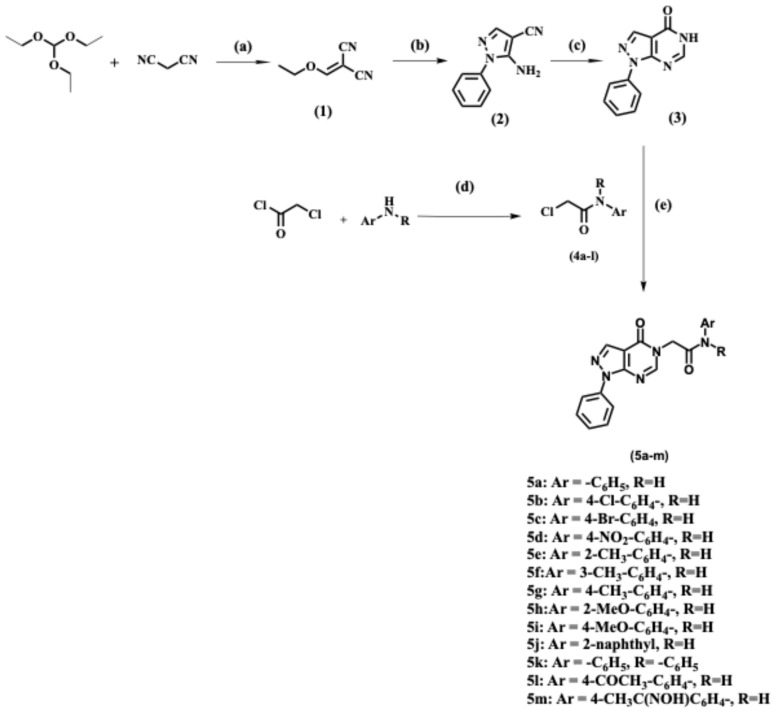
Synthesis of *N*-alkylated pyrazolo[3,4-*d*]pyrimidin-4(5*H*)-one derivatives **5a-m. Reagents and reaction conditions**: (**a**) acetic anhydride, reflux 4–5 h; (**b**) C_6_H_5_-NHNH_2_, EtOH, reflux 18 h; (**c**) formic acid, reflux 4–5 h; (**d**) appropriate aromatic amine, glacial acetic acid, chloroacetyl chloride, 0 °C (0.5 h), then, at room temperature (1 h), CH_3_COONa; (**e**) K_2_CO_3_, DMF, reflux, 8 h.

The sequence of reactions used to synthesize *N*-alkylated pyrazolo[3,4-*d*]pyrimidin-4(5*H*)-one derivatives **5a**-**m** is shown in Scheme 1. Compound (**1**) was prepared according to the previously reported procedure [40], starting with the reaction of triethyl orthoformate with malonitrile in the presence of acetic anhydride under reflux conditions. The above step was followed by the reaction with phenyl hydrazine, using ethanol as a solvent, under reflux conditions, to afford 5-amino-1-aryl-1*H*-pyrazole-4-carbonitriles (**2**) [41]. Cyclization was then achieved upon the reaction of compound (**2**) with formic acid under reflux, resulting in the required key intermediate (**3**) [41].

The synthesis of 2-chloro-*N*-phenylacetamide derivatives **4a-l** was performed by reacting the appropriate amine derivative with chloroacetyl chloride in glacial acetic acid solvent, in the presence of sodium acetate as a base, to provide the required intermediates **4a-l [42,43,44,45,46,47,48,49,50,51,52,53,54]**. The structural elucidation of the synthesized intermediates (**1–3**) and **4a-l** was based on their reported melting points.

Alkylation of the *NH* group of compounds (**3**) was achieved by refluxing compounds **4a-l** with anhydrous potassium carbonate as the base, using *N, N*-dimethylformamide (DMF) as the solvent, to afford the target compounds **5a-m**. The purity of these novel compounds was confirmed by IR, ^1^H NMR, ^13^C NMR, and elemental analysis methods (Figures S1–S24).

The ^1^H NMR spectra of compounds **5a-m** were characterized by the appearance of singlet signal at δ 11.10–9.84 ppm related to the N-H proton, a singlet signal for the pyrimidine-H at δ 8.59–8.41 ppm, another singlet signal for the pyrazole-H at δ 8.44–8.37 ppm, and a singlet signal of two protons related to the methylene protons (CO-CH_2_) at δ 5.13–4.74 ppm. At the same time, their ^13^C NMR spectra were characterized by the appearance of two characteristic peaks related to the two carbonyl carbons (CH_2_-C=O-N- of the amide) at δ 166.90–165.30 ppm and (C=O of pyrimidin-4-one ring) at δ 157.00–156.70 ppm, in addition to the methylene carbon attached to *N*-3 of the pyrimidinone ring (N-CH_2_-CO), which appeared at δ 49.0–48.30 ppm.

The ^1^H NMR spectrum of compound **5c**, as a representative example, showed one singlet proton for N-H at δ 10.52 ppm, one singlet proton for pyrimidine-H at δ 8.49 ppm, another singlet proton for pyrazole-H at δ 8.38 ppm, and a singlet signal related to the methylene protons (CO-CH_2_) at δ 4.93 ppm. Also, nine aromatic protons appeared at δ 8.07–7.42 ppm. The ^13^C NMR spectrum of compound **5c** showed two characteristic peaks at δ 166.01 ppm and δ 156.92 ppm, corresponding to the carbonyl groups (CH_2_-C=O-N- of the amide) and (C=O of the pyrimidin-4-one ring, respectively). Moreover, a characteristic peak appeared at δ 48.78 ppm related to the carbon of the methylene group attached to *N*-3 of the pyrimidinone ring (N-CH_2_-CO). Furthermore, the IR spectrum of compound **5c** showed a significant stretching band at 3313 cm^−1^, corresponding to the NH group. Two stretching bands appeared at 1678 and 1698 cm^−1^ due to the presence of two carbonyl groups: the C=O of the amide and the C=O of the pyrimidinone ring, respectively.

### 2.2. Evaluation of Biological Activities

#### 2.2.1. In Vitro Cyclooxygenase Inhibitory Assay

The final thirteen compounds (**5a-m**) were evaluated for their COX inhibitory activity on both human recombinant COX-1 and COX-2 isozymes using celecoxib as a reference compound [55,56]. The fluorescent inhibitor screening assay kit (Bio-Vision’s COX1/2 Inhibitor Screening Kit, Milpitas, CA, USA) was used for this assay. The results indicated that compounds **5a**, **5b**, **5c**, **5e**, **5f**, **5g**, **5h**, **5i**, and **5l** had no inhibitory activity on COX-1 and COX-2 isozymes at a concentration of 100 μM. On the other hand, compounds **5d**, **5j**, **5k**, and **5m** represented different percentages for both COX-1 and COX-2. The IC_50_ values of the tested compounds on both COX-1 and COX-2 isozymes were calculated, and the results are listed in Table 1.

The results revealed that most of the tested compounds exhibited a remarkable inhibition of the COX-2 isozyme, with IC_50_ values ranging from 0.26 to 0.58 μM, whereas COX-1 inhibition had IC_50_ values ranging from 6.40 to 57.31 μM. Compound **5k**, the diphenyl derivative of pyrazolo[3,4-*d*]pyrimidinone (R = -C_6_H_5_, Ar = -C_6_H_5_), exhibited comparable percent of inhibition against COX-2 isozyme to that of celecoxib (IC_50_ = 0.293 μM, SI = 98.70), with an IC_50_ value of 0.266 μM and SI = 95.75. Moreover, compounds **5d** and **5m** exhibited moderate selectivity toward COX-2, compared to celecoxib, with selectivity indices of 28.73 and 24.47, respectively. Although our assays determined IC_50_ values and selectivity, kinetic analyses were not performed. Future work will address this limitation by conducting detailed kinetic studies for the lead compound to define its inhibition mechanism.

#### 2.2.2. In Vivo Carrageenan-Induced Paw Edema

**Figure 2:**
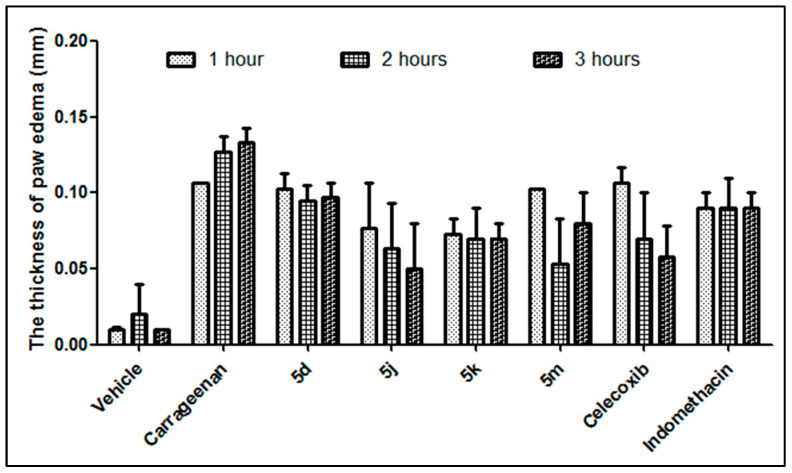
The effects of different compounds on carrageenan-induced rat paw edema. The values are presented as the mean ± SD, (n = 6). *p* < 0.05 compared with the carrageenan group (one-way ANOVA followed by the Tukey test).

Compounds **5d**, **5j**, **5k**, and **5m** were evaluated for their anti-inflammatory activity using the carrageenan-induced paw edema model in rats, using the dose of 50 mg/kg, as reported by Winter et al. [57]. After administering an intraplantar injection of 1% carrageenan to adult male mice (n = 6/group), paw swelling was measured at 1, 2, and 3 h. The mean changes in paw edema values are presented in Figure 2. Compared to the control, compounds **5j** (**R = H, Ar = 2-naphthyl**) and **5k** (**R = -C_6_H_5_, Ar = -C_6_H_5_**) showed better anti-inflammatory activity with a percentage of inhibition of paw edema of 62.41% and 54.89% at 3 h, respectively, which was higher than the paw edema inhibition induced by indomethacin after 3 h (32.33%). Compound **5m** (**R = H, Ar = 4-CH_3_C(NOH)C_6_H_4_**) also demonstrated strong anti-inflammatory activity at 2 h after carrageenan administration, with suppression of edema by 58.01%. The percentages of inhibition of paw edema are presented in Table 2.

**Figure 3:**
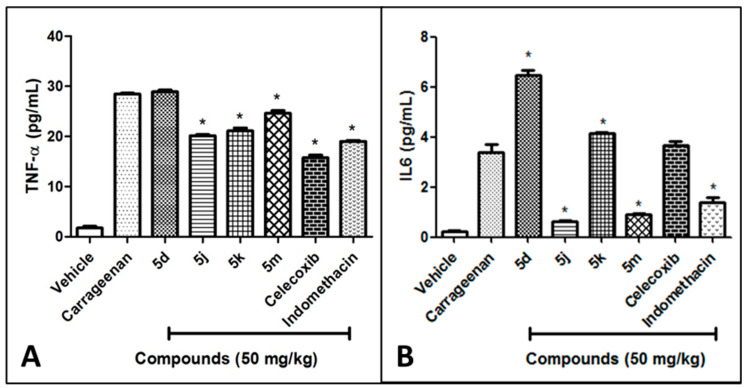
Effects of compounds on levels of (**A**) TNF-α and IL-6 (**B**) in the serum of carrageenan-treated mice at 3 h. The values are presented as the mean ± SD. * *p* < 0.05 compared with the carrageenan group (one-way ANOVA followed by the Tukey test).

To evaluate the anti-inflammatory mechanisms of these compounds, two key chemical mediators, TNF-α and IL-6, were assessed using enzyme-linked immunosorbent assay (ELISA). As shown in Figure 3A, carrageenan injection induced a remarkable increase in the concentration of TNF-α in serum. After treatment with the tested compounds (50 mg/kg), the TNF-α levels in serum were significantly reduced (*p* < 0.05) with compounds **5j, 5m**, and **5k,** compared to the control group. No significant differences in TNF-α were observed between compound **5d** (**R = H, Ar = 4-NO_2_-C_6_H_4_**) and the control group.

Similarly, carrageenan injection induced a significant increase in the concentration of the pro-inflammatory cytokine IL-6 in serum [58], which was significantly reduced (*p* < 0.05) in the groups treated with compounds **5j**, **5m**, and **5k** (Figure 3B). Compound **5j** (**R = H, Ar = 2-naphthyl**) demonstrated the highest inhibition of IL-6 levels, approaching normal levels, with no significant difference compared to the vehicle. Therefore, the anti-inflammatory activity of the tested compounds may hinge on their ability to suppress the pro-inflammatory TNF-α and IL-6 levels [59].

#### 2.2.3. Histopathological Investigation

**Figure 4:**
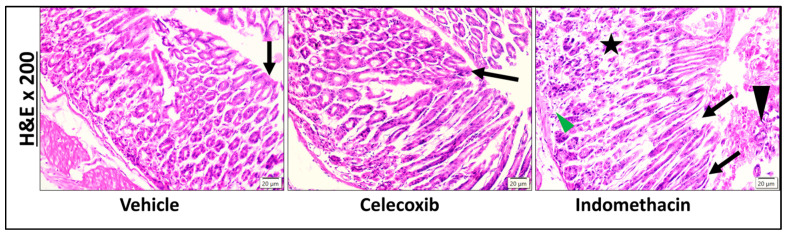
Photomicrographs by light microscopy illustrating stomach tissue stained with H&E for histopathological assessment.

**Figure 5:**
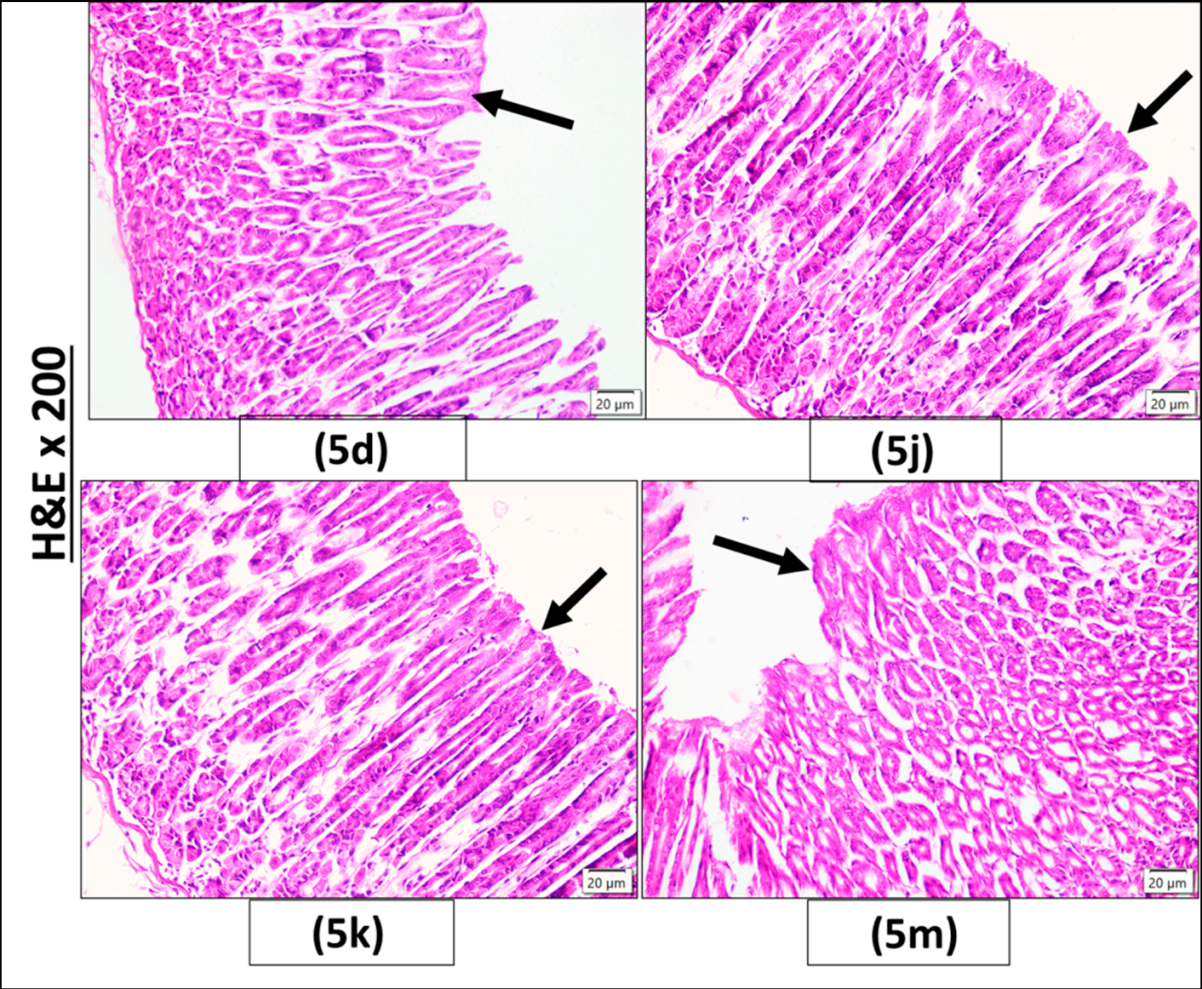
Photomicrographs of stomach tissue sections stained with H&E for histopathological assessment by light microscopy.

The results of the histopathological examination of the mice’s stomach sections stained with H&E are displayed in Figure 4 and Figure 5. The histopathological investigation displayed a normal gastric mucosal histology for these compounds and celecoxib. This ensured its gastrointestinal safety profile and potential therapeutic value. In contrast, the mice treated with indomethacin developed mucosal damage, sloughing, granulation tissue, and a lymphocytic infiltrate, indicating its potential for an ulcerative effect.

The control (vehicle) group and the celecoxib group exhibited a normal histological structure of the gastric mucosa, characterized by normal fundic gland morphology and intact surface mucosal epithelium (black arrow). However, the indomethacin group shows discontinuity of the surface mucosal lining (black arrow) with desquamated small cells having deep acidophilic cytoplasm and pyknotic nuclei (black arrowhead), loss of normal histological morphology of the gastric glands (black star), and inflammatory cellular infiltration (green arrowhead). (H&E ×200, scalebar; 20 µM).

Groups treated with compounds **5d**, **5j**, **5k**, and **5m** exhibited a normal histological structure of the gastric mucosa, characterized by normal fundic gland morphology and an intact surface mucosal epithelium (indicated by the black arrow). (H&E ×200, scale bar; 20 µM). From the abovementioned results, the value of the synthesized compounds as anti-inflammatory agents lies in their high safety for the gastric mucosa, similar to that of celecoxib.

### 2.3. Molecular Modelling Study

For molecular docking and molecular dynamics simulations, the crystal structures of human cyclooxygenase-1 (PDB ID: 3KK6) and cyclooxygenase-2 (PDB ID: 3LN1) were selected. These structures were chosen due to their high-resolution crystallographic data (2.75 Å and 2.40 Å, respectively) and the presence of the co-crystallized selective inhibitor—celecoxib—which provided reference ligands for grid box definition and comparative interaction analysis. Furthermore, both entries are widely used in COX-focused structure-based drug design, offering a well-established and validated framework for modelling studies. Importantly, these structures are free of artificial mutations or structural artifacts, ensuring accurate representation of the native enzyme conformations and active site architecture.

#### Target Identification

**Figure 6:**
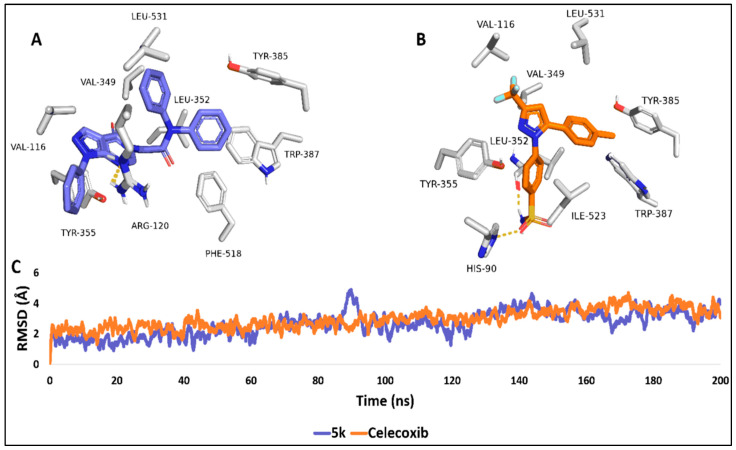
(**A**,**B**): Binding conformations of compound **5k** within the active site of human COX-1 (PDB ID: 3KK6), alongside the co-crystallized inhibitor celecoxib, both depicted as the most populated binding poses from a 200 ns molecular dynamics (MD) simulation. (**C**): RMSD profiles of **5k** and celecoxib throughout the 200 ns MD simulation.

The molecular docking and molecular dynamics (MD) simulation analyses were performed to investigate the binding interactions of compound **5k** (Figure 6A) and the co-crystallized inhibitor celecoxib (Figure 6B) within the active site of cyclooxygenase-1 (COX-1; PDB ID: 3KK6). The binding modes depicted correspond to the most populated poses derived from a 200-nanosecond molecular dynamics (MD) simulation, providing detailed insight into the stability and interaction profiles of both ligands.

In the binding pose of compound **5k** (Figure 6A), key interactions are observed with critical residues in the COX-1 active site. Specifically, **5l** forms a single hydrogen bond with ARG-120, while hydrophobic interactions with residues such as LEU-531, TRP-387 (π-π interaction), LEU-352, VAL-349, PHE-518, and TYR-355 (π-π interactions) contribute to the overall stability of the complex. The positioning of **5k** within the binding pocket highlights its effective engagement with the enzyme’s active site, albeit with a moderate affinity.

Conversely, celecoxib (Figure 6B), the co-crystallized reference inhibitor, exhibits a comparable binding orientation, forming notable hydrogen bonds with LEU-352 and establishing hydrophobic interactions with key residues such as ILE-523, VAL-116, and TYR-385. The positioning of HIS-90 within proximity to celecoxib suggests additional stabilization through electrostatic interactions. These interactions align well with the known pharmacophore of COX-1 inhibitors, indicating that celecoxib retains its expected binding behavior during the simulation.

The root mean square deviation (RMSD) analysis of the protein–ligand complexes over the 200 ns simulation (Figure 6C) further supports the stability of both ligand–enzyme systems. The RMSD profiles reveal that both compounds maintain a relatively stable interaction with COX-1, with celecoxib (orange line) demonstrating a slightly lower degree of structural fluctuation compared to **5k** (blue line). The fluctuations observed are within an acceptable range, indicating that both compounds maintain their binding orientations throughout the simulation. The average RMSD for both compounds was 3.49 Å and 3.31 Å, respectively.

The binding free energy (Δ*G*), as calculated via the molecular mechanics/Poisson–Boltzmann surface area (MM/PBSA) method, was determined to be −6.19 kcal/mol for **5k** and −6.59 kcal/mol for celecoxib. These values reflect moderate binding affinities, which are consistent with the in vitro inhibitory activity observed for both compounds.

**Figure 7:**
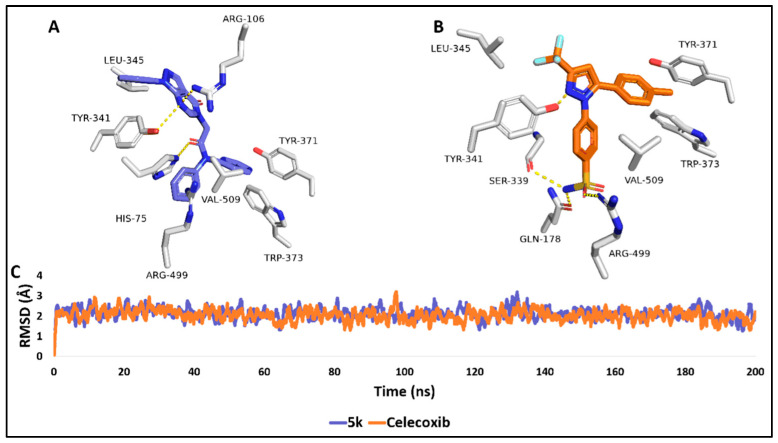
(**A**,**B**): Binding conformations of compound **5k** within the active site of human COX-2 (PDB ID: 3LN1), alongside the co-crystallized inhibitor celecoxib, both depicted as the most populated binding poses from a 200 ns MD simulation. (**C**): RMSD profiles of **5k** and celecoxib throughout the 200 ns MD simulation.

Similarly, molecular docking and molecular dynamics (MD) simulations for compound **5k** and the co-crystallized inhibitor celecoxib within the active site of cyclooxygenase-2 (COX-2; PDB ID: 3LN1) were conducted to evaluate their binding interactions and stability, with a comparative perspective to the previously analyzed COX-1 system. The binding modes presented in Figure 7A,B represent the most populated poses extracted from a 200 ns long MD simulation, providing key insights into the binding behaviors of both ligands within the COX-2 active site.

In Figure 7A, compound **5k** exhibits strong interactions with the COX-2 active site, particularly by forming hydrogen bonds with critical residues Arg-106, Tyr-341, and His-75. In addition, hydrophobic interactions with residues such as Leu-345, Val-509, and Trp-373 significantly contribute to the stabilization of the **5k-**COX-2 complex. Moreover, ARG-499 was involved in π-cation interaction with one of the terminal phenyl rings of **5k**. The binding pose of **5k** in COX-2 is comparable to that observed in COX-1, but with enhanced interaction networks, which likely accounts for its improved binding affinity.

Similarly, in Figure 7B, celecoxib forms crucial hydrogen bonds with residues ARG-499 and GLN-178 and exhibits strong hydrophobic interactions with TYR-371, TRP-373, and VAL-509. These interactions reinforce celecoxib’s known selectivity for COX-2, where the inhibitor’s orientation and interaction network indicate a more favorable binding affinity compared to COX-1.

The RMSD analysis (Figure 7C) shows that both ligands maintain stable interactions within COX-2 throughout the 200 ns MD simulation, with only minor fluctuations observed. Celecoxib (orange line) exhibits slightly lower RMSD values than **5k** (blue line), indicating marginally better structural stability, with average RMSDs of 2.08 Å and 2.19 Å, respectively. However, both ligands demonstrate overall good stability within the COX-2 binding pocket, comparable to their behavior in the COX-1 complex.

The calculated binding free energy (ΔG) using the MM/PBSA method reveals significantly stronger binding affinities for both compounds in COX-2 compared to COX-1. Compound **5k** exhibited a Δ*G* of −10.57 kcal/mol, while celecoxib demonstrated a ΔG of −10.19 kcal/mol. These substantially lower binding energy values correlate with the potent in vitro inhibitory activity observed for both **5k** and celecoxib against COX-2. The stronger binding interactions within COX-2 reflect the enhanced inhibitory potency of both compounds, making them highly effective in targeting COX-2 over COX-1.

To better understand the structure–activity relationships within the synthesized series, we extended the molecular modeling analysis to include compound **5c**, a representative weakly active analog (COX-2 IC_50_ > 100 µM), in parallel with the potent compound **5k**. The comparative docking results and 200 ns MD simulations revealed distinct differences in their binding modes and interaction profiles within the COX-2 active site (PDB: 3LN1).

Compound **5k**, characterized by a diphenylacetamide moiety at the N5 position, adopts a favorable orientation that maximizes hydrophobic surface engagement and aromatic stacking interactions. Notably, its terminal phenyl rings participate in π–π stacking with TRP-373 and TYR-341 and a π–cation interaction with ARG-499, which are critical determinants of COX-2 selectivity. Additionally, the extended hydrophobic interface of **5k** is well-aligned with the secondary pocket of COX-2, enhancing van der Waals contacts and contributing to its high binding affinity (Δ*G* = –10.57 kcal/mol).

In contrast, compound **5c**, bearing a monoaryl bromo-substituted acetamide group, showed a less optimal fit within the COX-2 binding cleft. Its truncated substitution pattern limited its ability to engage in key interactions with residues such as ARG-499, HIS-75, and TYR-341, and its binding conformation lacked the extensive aromatic surface area needed for stabilizing π-stacking. These structural limitations are consistent with its poor in vitro COX inhibition profile and were reflected in a higher RMSD drift (~4.1 Å) during the MD trajectory and a significantly weaker MM-PBSA binding free energy (Δ*G* = –6.02 kcal/mol).

Collectively, these findings underscore the importance of the diphenylacetamide motif in **5k** as a structural determinant of COX-2 selectivity and potency. This comparative analysis helps rationalize the inactivity of certain analogues and guides future scaffold optimization.

To gain deeper insight into protein flexibility and ligand stability, we evaluated the RMSF and hydrogen bond formation patterns over the 200 ns MD simulations. The RMSF plots revealed that binding of compound **5k** and celecoxib reduced residue-level fluctuations compared to the apo form of both COX-1 and COX-2, particularly in dynamic loop regions (residues 100–130 and 300–340), indicating conformational stabilization upon ligand binding (Figure S26).

In parallel, the number of hydrogen bonds formed between each ligand and the protein was monitored throughout the simulation. Compound **5k** exhibited stable hydrogen bonding in both COX isoforms, maintaining 1–2 persistent H-bonds per frame, which is comparable to the co-crystallized ligand celecoxib (Figure S27). This behavior is consistent with the observed binding free energy profiles and RMSD stability discussed earlier.

### 2.4. Predicted Physicochemical Properties and Toxicity Parameters

**Figure 8:**
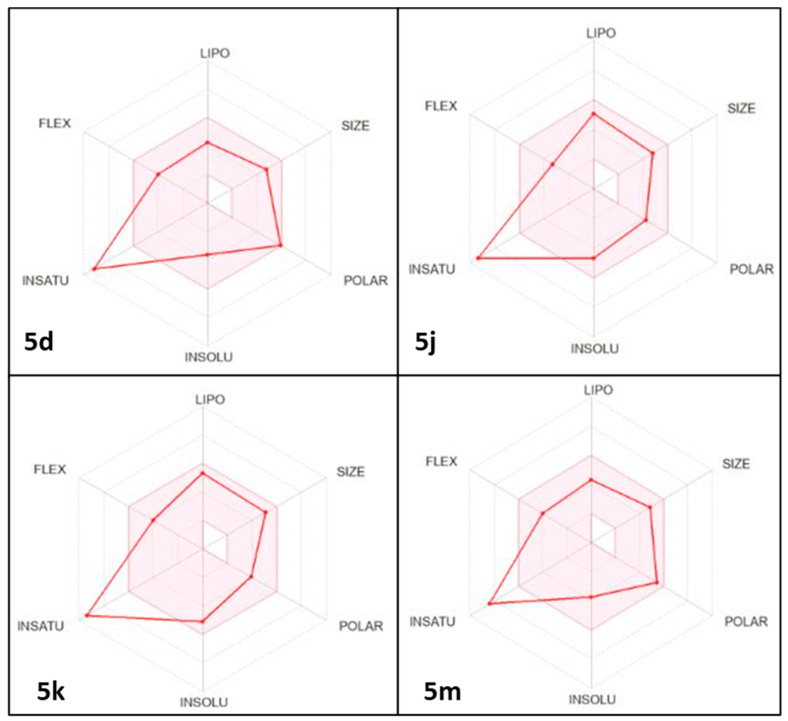
Rader model of compounds **5d**, **5j**, **5k**, and **5m**.

**Figure 9:**
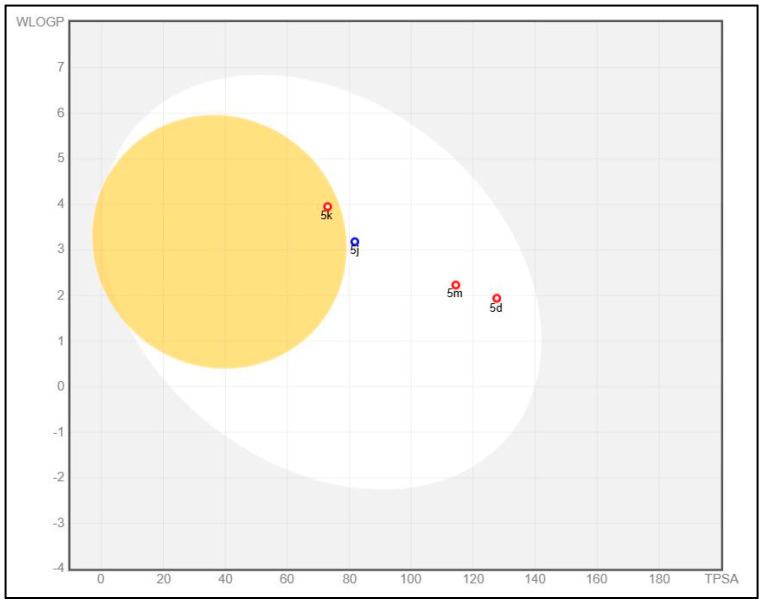
The BOILED-Egg model of compounds **5d**, **5j**, **5k**, and **5m**.

Promising drug candidates require a reasonable pharmacokinetic profile to be considered for clinical trials. Hence, the physicochemical and pharmacokinetic properties of the target compounds were predicted using Swiss ADME [60]. As illustrated in Table 3, Table 4, Table 5 and Table 6 and Figure 8 and Figure 9, all the target compounds were expected to exhibit high gastrointestinal absorption. Apart from compound **5k**, all target compounds were predicted to not cause central adverse effects as they were not predicted to pass through the blood–brain barrier. Consequently, compound **5k**, the most potent anti-inflammatory agent among the synthesized compounds, is anticipated to be significant in conditions such as multiple sclerosis, Alzheimer’s disease, Parkinson’s disease, traumatic brain injury, stroke, and meningitis, where inflammation occurs within the brain or spinal cord.

Compound **5j** was predicted to be resistant to Pgp efflux. The expected impact of the target drugs on CYP450 enzymes, specifically CYP1A2, CYP2C19, CYP2C9, CYP2D6, and CYP3A4, suggests a low likelihood of drug–drug interactions occurring. All the target compounds have molecular weights below 500 g/mol. All the target compounds have no violations in the Ghose filter. All the tested compounds have no violations in either the Veber or Egan filters. All the compounds have no violations in the Muegge filter. In terms of the Abbott bioavailability score, it was observed that all the target derivatives exhibit favorable oral absorption. The bioavailability radar model comprises six physicochemical properties: lipophilicity, polarity, size, flexibility, solubility, and saturation. The lipophilicity of all the target compounds is in an unacceptable range (XLOGP3 from −0.7 to +5.0). The size of all the target compounds falls within a range of 150 g/mol to 500 g/mol, and the polarity of all the target compounds is within a reasonable range, with a TPSA of 20 Å^2^ to 130 Å^2^. All the target compounds exhibited acceptable solubility, with Log S (ESOL) values ranging from -6 to 0. All the target compounds do not have a reasonable instauration degree (Fraction Csp3 range from 0.25 to 1.0). Finally, adequate flexibility was observed in all the target compounds, with fewer than nine rotatable bonds. To obtain a precise prediction of drug penetration in both the brain and the gastrointestinal tract, the BOILED-Egg approach is a reliable model to use [61]. It accomplishes this via calculating the lipophilicity (expressed in terms of WLOGP, yellow circle) and polarity (expressed in terms of TPSA, white circle) of the tested compounds Figure 9. The target compounds exhibited significant gastrointestinal absorption. In addition, it has been confirmed that all target compounds do not penetrate the blood–brain barrier, signifying their advantageous safety profile for the central nervous system.

## 3. Experimental Methods

### 3.1. Chemistry

#### 3.1.1. General Procedure for the Synthesis of 2-(4-Oxo-1-Phenyl-1,4-Dihydro-5H-Pyrazolo[3,4-*d*]Pyrimidin-5-yl)-N-Phenylacetamide Derivatives (**5a-m**)

To a stirred mixture of compound **3** (0.424 g, 2.0 mmol) and potassium carbonate (0.6905 mmol) in DMF, the appropriate alkylating agent **4a-l** (1.2 mmol) was added. The solution was refluxed for 8 h. Then, the reaction mixture was poured onto crushed ice, and the reaction progress was monitored by TLC (eluent: DCM/Ethanol, 9.5:0.5). The formed precipitate was filtered off, washed with water, dried, and recrystallized from ethanol to obtain the final compounds (**5a-m**).

##### 2-(4-Oxo-1-Phenyl-1,4-Dihydro-5H-Pyrazolo[3,4-*d*]Pyrimidin-5-yl)-N-Phenyl Acetamide. (**5a**)

White powder, yield: (0.45g, 69%), m.p.: 260–262 °C; IR (KBr, cm^−1^): 3308 (NH), 1693, 1669 (C=O); ^1^H NMR (400 MHz, DMSO-*d*_6_): δ (ppm) 10.48 (s, IH, N-H); 8.52 (s, 1H, pyrimidine-H); 8.41 (s, 1H, pyrazole-H), 8.07 (d, *J* = 8.0 Hz, 2H, Ar-H), 7.62–7.58 (m, 4H, Ar-H), 7.44 (t, *J* = 8.0 Hz, 1H, Ar-H), 7.36–7.32 (m, 2H, Ar-H), 7.09 (t, *J* = 8.0 Hz, 1H, Ar-H), 4.93 (s, 2H, CH_2_); ^13^C NMR (100 MHz, DMSO-*d*_6_):δ (ppm):165.79, 156.86, 152.54, 151,80, 139.06, 138.56, 136.59, 129.78, 129.37, 127.74, 124.07, 122.24, 119.55, 107.18, 48.71; anal. calcd. for C_19_H_15_N_5_O_2_: C%, 66.08; H%, 4.38; N%, 20.28; found: C%, 66.29; H%, 4.42; N%, 20.57.

##### *N*-(4-Chlorophenyl)-2-(4-Oxo-1-Phenyl-1,4-Dihydro-*5H*-Pyrazolo[3,4-*d*]Pyrimidin-5-yl) Acetamide. (**5b**)

Yellowish-white powder, yield: 0.47g (62.6%), m.p.: 262–264 °C; IR (KBr, cm^−1^): 3316 (NH), 1613, 1669 (C=O); ^1^H NMR (400 MHz, DMSO-*d*_6_): δ (ppm) 10.64 (s, IH, N-H); 8.51 (s, 1H, pyrimidine-H); 8.40 (s, 1H, pyrazole-H), 8.06 (d, *J =* 8.0 Hz, 2H, Ar-H); 7.64–7.58 (m, 4H, Ar-H), 7.45 (d, *J* = 8.0 Hz, 1H, Ar-H), 7.40 (d, *J* = 8.0 Hz, 2H, Ar-H), 4.92 (s, 2H, CH_2_); ^13^C NMR (100 MHz, DMSO-*d*_6_): δ (ppm):165.99, 156.92, 152.88, 152.42, 151,79, 138.47, 137.88, 136.53, 129.78, 129.26, 127.84, 122.33, 121.27, 107.09, 48.73; anal. calcd. for C_19_H1_4_ClN_5_O_2_: C%, 60.09; H%, 3.72; Cl%, 9.33; N%, 18.44; found: 60.23; H%, 3.64; N%, 18.69.

##### *N*-(4-Bromophenyl)-2-(4-Oxo-1-Phenyl-1,4-Dihydro-*5H*-Pyrazolo[3,4-*d*]Pyrimidin-5-yl) Acetamide. (**5c**)

White powder, yield: 0.55g (65.5%), m.p.: 230–232 °C; IR (KBr, cm^−1^): 3313 (NH), 1678, 1698 (C=O); ^1^HNMR (400 MHz, DMSO-*d*_6_):δ (ppm)10.52 (s, IH, N-H); 8.49 (s, 1H, pyrimidine-H); 8.38 (s, 1H, pyrazole-H), 8.07 (d, *J* = 8.0 Hz, 2H, Ar-H), 7.61–7.53 (m, 4H, Ar-H), 7.52–7.45 (m, 2H, Ar-H), 7.42 (d, 1H, *J* = 8.0 Hz, Ar-H), 4.93 (s, 2H, CH_2_); ^13^C NMR (100 MHz, DMSO-*d*_6_):δ (ppm):166.01, 156.92, 152.42, 151.74, 138.43, 138.28, 136.55, 132.20, 129.80, 127.85, 122.31, 121.57, 115.78, 107.08, 48.78; anal. calcd. for C_19_H_14_BrN_5_O_2_: C%, 53.79; H%, 3.33; N%, 16.51; found: C%, 54.05; H%, 3.59; N%, 16.81.

##### *N*-(4-Nitrophenyl)-2-(4-oxo-1-phenyl-1,4-dihydro-*5H*-pyrazolo[3,4-*d*]pyrimidin-5-yl) Acetamide. (**5d**)

Yellowish powder, yield: 0.48g (62.3%), m.p:180–182 °C; IR (KBr, cm^−1^): 3358 (NH), 1617, 1682 (C=O); ^1^H NMR (400 MHz, DMSO-*d*_6_): δ (ppm) 11.10 (s, IH, N-H); 8.53 (s, 1H, pyrimidine-H); 8.41 (s, 1H, pyrazole-H), 8.26 (d, *J* = 8.0 Hz, 2H, Ar-H), 8.06 (d, *J* = 8.0 Hz, 2H, Ar-H), 7.86–7.83 (m, 2H, Ar-H), 7.58–7.62 (m, 2H, Ar-H), 7.44 (t, *J* = 8.0 Hz, 1H, Ar-H), 4.99 (s, 2H, CH_2_); ^13^C NMR (100 MHz, DMSO-*d*_6_):δ (ppm): 166.90, 156.83, 152.41, 151.75, 145.07, 142.99, 138.49, 136.62, 129.80, 127.80, 125.62, 122.27, 119.39, 107.11, 49.01; anal. calcd. for C_19_H_14_N_6_O_4_: C%, 58.46; H%, 3.62; N%, 21.53; found: C%, 58.62; H%, 3.71; N%, 21.80.

##### 2-(4-Oxo-1-Phenyl-1,4-Dihydro-*5H*-Pyrazolo[3,4-*d*]Pyrimidin-5-yl)-*N*-(o-Tolyl) Acetamide. (**5e**)

White powder, yield: 0.6g (85%), m.p.: 238–240 °C; IR (KBr, cm^−1^): 3253 (NH), 1659, 1698 (C=O); ^1^H NMR (400 MHz, DMSO-*d*_6_): δ (ppm) 9.84 (s, IH, N-H) 8.53 (s, 1H, pyrimidine-H); 8.41 (s, 1H, pyrazole-H), 8.06 (d, *J* = 8.0 Hz, 2H, Ar-H), 7.61–7.57 (m, 2H, Ar-H), 7.45–7.39 (m, 2H, Ar-H), 7.24 (d, 1H, *J* = 8.0 Hz, Ar-H), 7.19–7.09 (m, 2H, Ar-H), 4.97 (s, 2H, CH_2_) 2.55 (s, 3H, CH_3_); ^13^C NMR (100 MHz, DMSO-*d*_6_):δ (ppm):166.02, 156.86, 152.53, 151.84, 138.60, 136.57, 136.19, 132.36, 130.87, 129.75, 127.71, 126.47, 125.97, 125.41, 122.22, 107.22, 48.53, 18.29; anal. calcd. for C_19_H_15_N_5_O_2_: C%, 66.84; H%, 4.77; N%, 20.49; found: C%, 66.97; H%, 4.82; N%, 19.67.

##### 2-(4-Oxo-1-Phenyl-1,4-Dihydro-*5H*-Pyrazolo[3,4-*d*]Pyrimidin-5-yl)-*N*-(m-Tolyl) Acetamide. (**5f**)

White powder, yield: 0.49 g (59%), m.p.: 248–250 °C; IR (KBr, cm^−1^): 3319 (NH), 1618, 1668 (C=O); ^1^H NMR (400 MHz, DMSO-*d*_6_): δ (ppm) 10.47 (s, IH, N-H) 8.52 (s, 1H, pyrimidine-H); 8.40 (s, 1H, pyrazole-H), 8.07 (d, *J* = 8.0 Hz, 2H, Ar-H), 7.61–7.57 (m, 2H, Ar-H), 7.45–7.39 (m, 3H, Ar-H), 7.21 (t, *J* = 8.0 Hz, 1H, Ar-H), 6.90 (d, *J* = 8.0 Hz, 1H, Ar-H), 4,94 (s, 2H, CH_2_) 2.28 (s, 3H, CH_3_); ^13^C NMR (100 MHz, DMSO-*d*_6_): δ (ppm):165.74, 156.89, 152.51, 151,78, 138.98, 138.57, 138.52, 136.55, 129.17, 129.17, 127.75, 124.78, 122.23, 120.14, 116.78, 107.15, 48.73, 21.61; anal. calcd. for C_20_H_17_N_5_O_2_: C%, 66.84; H%, 4.77; N%, 19.49; found: C%, 67.05; H%, 4.93; N%, 19.70.

##### 2-(4-Oxo-1-Phenyl-1,4-Dihydro-*5H*-Pyrazolo[3,4-*d*]Pyrimidin-5-yl)*-N-*(p-Tolyl) Acetamide. (**5g**)

White powder, yield: 0.42g (50.6%), m.p.: 275–277 °C; IR (KBr, cm^−1^): 3317 (NH), 1615, 1683 (C=O); ^1^H NMR (400 MHz, DMSO-*d*_6_): δ (ppm) 10.49 (s, IH, N-H) 8.50 (s, 1H, pyrimidine-H); 8.38 (s, 1H, pyrazole-H), 8.05 (d, *J* = 8.0 Hz, 2H, Ar-H), 7.61–7.57 (m, 2H, Ar-H), 7.48 (d, *J* = 8.0 Hz, 2H, Ar-H), 7.45–7.41 (m, 1H, Ar-H), 7.13 (d, *J* = 8.0 Hz, 2H, Ar-H), 4,92 (s, 2H, CH_2_) 2.25 (s, 3H, CH_3_); ^13^C NMR (100 MHz, DMSO-*d*_6_):δ (ppm): 165.56, 156.88, 152.50, 151,80, 138.57, 136.59, 136.50, 133.0, 129.70, 129.65, 127.67, 122.18, 119.64, 107.18, 48.67, 20.86; anal. calcd. for C_20_H_17_N_5_O_2_: C%, 66.84; H%, 4.77; N%, 19.49; found: C%, 67.06; H%, 4.93; N%, 19.65.

##### *N*-(2-Methoxyphenyl)-2-(4-Oxo-1-Phenyl-1,4-Dihydro-*5H*-Pyrazolo[3,4-*d*]Pyrimidin-5-yl) Acetamide. (**5h**)

Yellowish-white powder, yield: 0.65 g (87%), m.p.: 210–212 °C; IR (KBr, cm^−1^): 3392 (NH) 1602, 1695 (C=O);^1^H NMR (400 MHz, DMSO-*d*_6_): δ (ppm) 10.47 (s, IH, N-H) 8.51 (s, 1H, pyrimidine-H); 8.40 (s, 1H, pyrazole-H), 8.06 (d, *J* = 8.0 Hz, 2H, Ar-H), 7.62–7.85 (m, 2H, Ar-H), 7.44 (t, *J* = 8.0 Hz, 1H, Ar-H), 7.31–7.22 (m, 2H, Ar-H), 7.11 (d, *J* = 8.0 Hz, 1H, Ar-H) 6.68–6.65 (m, 1H, Ar-H), 4.91 (s, 2H, CH_2_), 3.72 (s, 3H, O-CH_3_); ^13^C NMR (100 MHz, DMSO-*d*_6_):δ (ppm):166.09, 156.83, 152.62, 151.82, 150,03, 138.59, 136.56, 130.55, 129.75, 127.71, 127.34, 125.13, 122.23, 120.76, 111.85, 107.18, 56.22, 48.70; anal. calcd. for C_20_H_17_N_5_O_3_: C%, 63.99; H%, 4.56; N%, 18.66; found: C%, 64.17; H%, 4.68; N%, 18.92.

##### *N*-(4-Methoxyphenyl)-2-(4-Oxo-1-Phenyl-1,4-Dihydro-*5H*-Pyrazolo[3,4-*d*]Pyrimidin-5-yl) Acetamide. (**5i**)

Yellowish-white powder, yield: 0.65 g (87%), m.p.: 259–261 °C; IR (KBr, cm^−1^):3314 (NH), 1615, 1677 (C=O); ^1^H NMR (400 MHz, DMSO-*d*_6_): δ (ppm) 10.36 (s, IH, N-H) 8.51 (s, 1H, pyrimidine-H); 8.40 (s, 1H, pyrazole-H), 8.06 (d, *J* = 8.0 Hz, 2H, Ar-H), 7.62–7.58 (m, 2H, Ar-H), 7.51 (d, *J* = 8.0 Hz, 2H, Ar-H), 7.44 (t, *J* = 8.0 Hz, 1H, Ar-H), 6.91 (d, *J* = 8.0 Hz, 2H, Ar-H), 4.89 (s, 2H, CH_2_), 3.75 (s, 3H, O-CH_3_); ^13^C NMR (100 MHz, DMSO-*d*_6_):δ (ppm):165.25, 156.85, 155.85, 152,58, 151.79, 138.54, 136.60, 132.19, 129.80, 127.75, 122.24, 121.05, 114.46, 107.17, 55.62, 48.56; anal. calcd. for C_20_H_17_N_5_O_3_ C%, 63.99; H%, 4.56; N%, 18.66; found: C%, 64.21; H%, 4.73; N%, 18.94.

##### *N*-(Naphthalen-2-yl)-2-(4-Oxo-1-Phenyl-1,4-Dihydro-5H-Pyrazolo[3,4-*d*]Pyrimidin-5-yl) Acetamide. (**5j**)

Greyish powder, yield: 0.45g (57.7%), m.p.:245–247 °C; IR (KBr, cm^−1^): 3255 (NH), 1661, 1715 (C=O); ^1^H NMR (400 MHz, DMSO-*d*_6_): δ (ppm) 10.45 (s, IH, N-H); 8.59 (s, 1H, pyrimidine-H); 8.44 (s, 1H, pyrazole-H), 8.19 (d, *J* = 12.0 Hz, 1H, Ar-H), 8.07 (d, *J* = 8.0 Hz, 2H, Ar-H), 7.97 (d, *J* = 8.0 Hz, 1H, Ar-H), 7.81 (d, *J* = 8.0 Hz, 1H, Ar-H), 7.70 (d, *J* = 8.0 Hz, 1H, Ar-H), 7.63–7.57 (m, 4H, Ar-H) 7.51 (t, *J* = 8.0Hz, 1H, Ar-H) 7.44 (t, *J* = 8.0 Hz, 1H, Ar-H) 5.13 (s, 2H, CH_2_); ^13^C NMR (100 MHz, DMSO-*d*_6_):δ (ppm):166.83, 157.01, 152.54, 151.84, 138.53, 136.60, 134.20, 133.41, 129.78, 128.63, 127.77, 126.64, 126.05, 123.24, 122.27, 107.24, 48.81; anal. calcd. for C_23_H_17_N_5_O_2_: C%, 69.86; H%, 4.33; N%, 17.71; found: C%, 70.08; H%, 4.51; N%, 17.94.

##### 2-(4-Oxo-1-Phenyl-1,4-Dihydro-5*H*-Pyrazolo[3,4-*d*]Pyrimidin-5-yl)-*N, N*-Diphenyl Acetamide. (**5k**)

White powder, yield: 0.49g (59%), m.p.: 199–201 °C; IR (KBr, cm^−1^); 1585,1684 (C=O); ^1^H NMR (400 MHz, DMSO-*d*_6_): δ (ppm); 8.41 (s, 1H, pyrimidine-H); 8.37 (s, 1H, pyrazole-H), 8.04 (d, *J* = 8.0 Hz, 2H, Ar-H), 7.68–7.56 (m, 5H, Ar-H), 7.44–7.41 (m, 8H, Ar-H) 4.74 (s, 2H, CH_2_); ^13^C NMR (100 MHz, DMSO-*d*_6_):δ (ppm):166.72, 156.66, 152.36, 151,76, 138.53, 136.50, 129.74, 127.74, 127.08, 127.07, 122.28, 107.1, 48.34; anal. calcd. for C_25_H_19_N_5_O_2_: C%, 71.25; H%, 4.54; N%, 16.62; found: C%, 71.43; H%, 4.61; N%, 16.89.

##### *N*-(4-Acetylphenyl)-2-(4-Oxo-1-Phenyl-1,4-Dihydro-5*H*-Pyrazolo[3,4-*d*]Pyrimidin-5-yl) Acetamide. (**5l**) [62]

Yellowish-white powder, yield: 0.35 g (45%), m.p.: 228–230 °C; reported m.p.: 234.7–237.9 °C [62]. IR (KBr, cm^−1^): 3281 (NH) 1601, 1676 (C=O);^1^H NMR (400 MHz, DMSO-*d*_6_): δ (ppm) 10.86 (s, IH, N-H); 8.51 (s, 1H, pyrimidine-H); 8.39 (s, 1H, pyrazole-H), 8.05 (d, *J* = 8.0 Hz, 2H, Ar-H), 7.96 (d, *J* = 8.0 Hz, 2H, Ar-H), 7.73 (d, *J* = 8.0 Hz, 2H, Ar-H), 7.62–7.85 (m, 2H, Ar-H), 7.45 (t, *J* = 8.0 Hz, 1H, Ar-H) 4.96 (s, 2H, CH_2_), 2.5 (s, 3H, CH_3_); ^13^C NMR (100 MHz, DMSO-*d*_6_): δ (ppm): 196.96, 166.44, 156.84, 152.46, 151.81, 143.28, 138.57, 136.58, 132.59, 130.04, 129.77, 127.76, 122.27, 118.94, 107.15, 48.86, 26.87; anal. calcd. for C_21_H_17_N_5_O_3_: C%, 65.11; H%, 4.42; N%, 18.08, found: C%, 65.29; H%, 4.53; N%, 18.30.

##### *N*-(4-(1-(Hydroxyimino)Ethyl)Phenyl)-2-(4-Oxo-1-Phenyl-1,4-Dihydro-5*H*-Pyrazolo[3,4-*d*]Pyrimidin-5-yl) Acetamide. (**5m**) [62]

White powder, yield: 0.29g (70.7%), m.p.: 250–252 °C; reported m.p.: 256.2–259.4 °C **[62]**; IR (KBr, cm^−1^): 3731 (OH), 3333 (NH), 1689 (C=O); ^1^H NMR (400 MHz, DMSO-*d*_6_): δ (ppm) 11.10 (s, IH, N-OH); 10.59 (s, IH, N-H) 8.51 (s, 1H, pyrimidine-H); 8.40 (s, 1H, pyrazole-H), 8.06 (d, *J* = 8.0 Hz, 2H, Ar-H), 7.65–7.58 (m, 6H, Ar-H), 7.44 (t, *J* = 8.0 Hz, 1H, Ar-H), 4.93 (s, 2H, CH_2_), 2.13 (s, 3H, CH_3_); ^13^C NMR (100 MHz, DMSO-*d*_6_):δ (ppm): 165.87, 156.85, 152.85, 152,50, 151.81, 139.34, 138.57, 136.57, 132.69, 129.76, 127.74, 126.62, 122.26, 119.31, 107.16, 48.72, 11.80; anal. calcd. for C_21_H_18_N_6_O_3_: C%, 62.68; H%, 4.51; N%, 20.88; found: C%, 62.69; H%, 4.65; N%, 21.14.

### 3.2. Biology

#### 3.2.1. In Vitro Cyclooxygenase (COX) Inhibition Assay

The tested compounds and celecoxib were evaluated for their ability to inhibit COX-1 and COX-2 isozymes using the COX Inhibitor Screening Kit (Fluorometric) from BioVision (Milpitas, CA, USA; Catalog No. K547), according to the manufacturer’s instructions [63]. Briefly, the peroxidase activity of the compounds was evaluated through a fluorogenic substrate that emits a fluorescent signal (Ex/Em = 535/587 nm) proportional to the enzyme activity. Test compounds, dissolved in DMSO, and were pre-incubated with the enzymes at 37 °C for 5 min before initiating the reaction with 1 mM arachidonic acid under light-protected conditions. The fluorescence was measured using a multi-well spectrophotometer (fluorescence plate reader). Indomethacin and celecoxib were used as positive controls for COX-1 and COX-2, respectively. The percentage inhibition was calculated by comparing the absorbance values of the compounds to the vehicle control.

#### 3.2.2. In Vivo Carrageenan-Induced Paw Edema

Eighty-four male BALB/c mice, aged 12 weeks, were procured from the Animal Facility of Sohag University. The Animal Ethics Committee of IACUC approved the protocol, approval number 12-3-1/2025-01. The previously described protocol by Winter et al. [64] was followed to perform the carrageenan-induced paw edema test, investigating the anti-inflammatory activity of the compounds relative to celecoxib and indomethacin as reference standards. To induce acute inflammation, 20 μL of carrageenan (1% *w*/*v*) was injected into the sub-plantar region of the right hind paw 30 min after oral administration of the compounds and the positive controls (50 mg/kg) in 1% carboxymethylcellulose (CMC) in distilled water. The control group received the vehicle. The thickness of the paw edema was measured before the induction of inflammation and then at intervals of one hour (1, 2, and 3 h) after carrageenan injection. The percentage of inhibition in the paw edema of the positive control and the compound-treated groups relative to the negative control was calculated using the formula: Inhibition (%) = (Control − Treated)/Control × 100.

#### 3.2.3. ELISA Assay for Tumor Necrosis Factor-α (TNF-α) and Interleukin-6 (IL-6)

All mice were sacrificed under anesthesia using ketamine/xylazine (50/15 mg/kg). Blood was collected for the ELISA assays to determine the levels of cytokines TNF-α and IL-6 using the Rat IL6 (Interleukin 6) ELISA Kit and Rat TNFα ELISA Kit (MyBioSource, San Diego, CA, USA), in accordance with the manufacturer’s instructions. The quantitative sandwich enzyme immunoassay technique is the fundamental principle of the TNF-α assay. A microplate was pre-coated with an antibody specific for TNF-α. Standards and samples were pipetted into the wells, and the immobilized antibody bound to any TNF-α that was present. A biotin-conjugated antibody that was specific for TNF-α was introduced to the wells after any unbound substances were removed. Horseradish peroxidase (HRP) conjugated with avidin was introduced to the wells subsequent to the cleaning process. After a wash to eliminate any unbound avidin–enzyme reagent, a substrate solution was introduced to the wells, and the color developed in proportion to the quantity of TNF-α bound in the initial step. The color development was halted, and the color intensity was quantified. Additionally, the Sandwich enzyme immunoassay is the test principle utilized for IL-6. The microtiter plates included in this preparation were pre-coated with an antibody that was specific to Rat IL6. A biotin-conjugated antibody specific to Rat IL6 was then added to the appropriate microtiter plate wells along with standards or samples. Subsequently, each microplate well was incubated with avidin conjugated to horseradish peroxidase (HRP). The only wells exhibited a change in color were those that contain Rat IL6, biotin-conjugated antibody, and enzyme-conjugated avidin after the TMB substrate solution was added. The addition of sulfuric acid solution terminated the enzyme–substrate reaction, and the color change was observed spectrophotometrically at a wavelength of 450 nm ± 10 nm. The concentration of Rat IL6 in the samples was subsequently determined by comparing the OD of the samples to the standard curve.

#### 3.2.4. Histopathological Examination

The stomachs were also dissected for histopathological examination to assess the degree of inflammatory response produced in the gastric layers using celecoxib as a reference drug. Stomach tissue sections were fixed using 10% buffered neutral formalin, and the stomach tissue samples were processed and stained by H&E [64]. The stained sections were examined and photographed using light microscopy (Olympus CX 43, Tokyo, Japan)

#### 3.2.5. Animals

Twelve-week-old male mice (120–150 gm) were obtained from the Animal Facility of Sohag University, Sohag, Egypt. The animals were housed together according to treatment randomization and subjected to a 12 h:12 h light–dark cycle in a temperature-controlled environment and maintained on a standard chow diet and water ad libitum.

#### 3.2.6. Blood Collection

Blood was collected immediately following the decapitation of mice, performed in accordance with the institutional animal care and use committee (IACUC) guidelines. After decapitation, whole blood was allowed to flow freely and was collected directly into EDTA and heparinized tubes. The whole blood was centrifuged at 3000× *g* for 10 min at 4 °C, and the plasma was carefully collected and stored at –80 °C until further analysis.

#### 3.2.7. Ethical Approval

All experimental protocols were accepted by the Sohag Institutional Animal Care and Use Committee (IACUC approval #: 12-3-1/2025-01)

#### 3.2.8. Statistical Analysis

Data were expressed as the mean and SD (n = 6). Statistical significance was determined using two-way ANOVA, with *p*-values less than 0.05 considered significant.

### 3.3. Molecular Docking

Molecular docking was carried out using AutoDock Vina 4.2, employing the rigid protein–flexible ligand protocol. The crystal structures of human COX-1 (PDB ID: 3KK6) and COX-2 (PDB ID: 3LN1) were prepared by removing co-crystallized ligands and water molecules, adding polar hydrogens, and assigning Gasteiger charges using AutoDock Tools.

The docking grid boxes were defined around the binding site of the co-crystallized ligands with the following parameters: for COX-1: grid center at (x = –19.67, y = 49.71, z = 41.53) with box dimensions of 28 × 28 × 28 Å; for COX-2: grid center at (x = 18.65, y = –53.12, z = 53.67) with box dimensions of 30 × 30 × 30 Å.

The exhaustiveness parameter was set to 16 to ensure thorough conformational sampling. Binding poses were ranked based on docking affinity scores, and the most populated poses from the MD simulations were used for further analysis.

The root mean square deviation (RMSD) threshold for ligand-to-binding site shape matching was set at 2.0 Å. The interaction energies were determined using the CHARMM force field (v. 1.02), with a non-bonded cutoff distance of 10.0 Å and a distance-dependent dielectric. An energy grid extending 5.0 Å from the binding site was established. Editing and visualization of the generated binding poses were performed using PyMOL software [65].

#### 3.3.1. Molecular Dynamics Simulation

MD simulations were conducted using NAMD 2.9 software, which employs the CHARMM36 force field. Protein systems were constructed using the QwikMD toolkit of VMD software [66], ensuring the protein structures had no missing hydrogens, setting the protonation states of the amino acid residues to pH 7.4, and removing co-crystallized water molecules. The structures were then embedded in an orthorhombic box of TIP3P water with 0.15 M Na^+^ and Cl^−^ ions in a 20 Å solvent buffer. The systems were energy minimized and equilibrated for 5 nanoseconds. The parameters and topologies of the ligands were calculated using the VMD plugin Force Field Toolkit 1.02 (ffTK). These generated parameters and topology files were loaded into VMD [67] to read the protein–ligand complexes accurately and conduct the simulation steps.

#### 3.3.2. Binding Free Energy Calculations

The binding free energy of the docked complex was calculated using the Molecular Mechanics Poisson–Boltzmann Surface Area (MM-PBSA) method, as implemented in the MMPBSA.py module of AMBER18 [68]. A total of 100 frames from the trajectories were processed, and the system’s net energy was estimated using the following equation:

ΔG Binding = ΔG Complex − ΔG Receptor − ΔG Inhibitor.

Each term in the equation involves calculating multiple energy components, including van der Waals energy, electrostatic energy, internal energy from molecular mechanics, and the polar contribution to solvation energy.

### 3.4. Physicochemical and Pharmacokinetic Prediction

The physicochemical characteristics and pharmacokinetics were predicted using Swiss ADME, a freely accessible service offered by the Swiss Institute of Bioinformatics [69]. The BOILED Egg graph illustrates the correlation between TPSA and WLOGP. The white region represents the highest probability of gastrointestinal absorption, while the yolk region indicates the greatest likelihood of BBB permeability. Lipophilicity is measured using the consensus log Po/w, which SwissADME calculates. The number represents the mean of five log *p*-values estimated by different publicly accessible models, specifically XLOGP3, MLOGP, SILICOS-IT, iLOGP, and their proprietary model WLOGP, which is also employed in the BOILED Egg plot. The bioavailability radar consists of six specific physicochemical properties: lipophilicity (ranging from −0.7 to +5.0 for XLOGP3), size (ranging from 150 g/mol to 500 g/mol for MV), polarity (ranging from 20 Å^2^ to 130 Å^2^ for TPSA), insolubility (ranging from 0 to 6 for Log S (ESOL)), unsaturation (ranging from 0.25 to 1.0 for Fraction Csp3), and flexibility (ranging from 0 to more than 9 for the number of rotatable bonds). The central pink hexagon represents the optimal range for all six criteria. The Lipinski filter is used to evaluate the drug-like properties of synthesized compounds. The evaluation considers the following criteria: The molecular weight (MW) must not exceed 500. The MLOGP value, which measures lipophilicity, must not exceed 4.15. The number of nitrogen or oxygen atoms must not exceed 10, and the number of NH or OH groups must not exceed 5.

## 4. Conclusions

In summary, 13 new hybrids of pyrazolo[3,4-*d*]pyrimidinone (**5a-m**) were synthesized and evaluated for their inhibitory activity toward COX-1 and COX-2 isozymes. Compared to celecoxib, the synthesized compounds exhibited remarkable inhibition of the COX-2 isozyme relative to COX-1 inhibition. Furthermore, in vivo carrageenan-induced rat paw edema tests proved that compounds **5j** and **5k** demonstrated significant anti-inflammatory efficacy, exhibiting greater suppression percentages of induced paw edema than indomethacin and comparable to celecoxib, with inhibition rates of 62.41% and 54.89% at 3 h, respectively, while maintaining excellent safety profiles with preserved gastric tissue integrity. Compound **5k** emerged as the most promising anti-inflammatory agent among the tested compounds with an IC_50_ value of 0.266 μM and a selectivity index (SI) of 95.8 for COX-2 relative to COX-1, compared to celecoxib (IC_50_ (COX-2) = 0.29 μM, SI = 98.70). Mechanistic studies demonstrated that the anti-inflammatory efficacy of the target compounds was associated with a substantial decrease in the serum levels of the pro-inflammatory cytokines TNF-α and IL-6. The tested compounds displayed a high safety profile with normal stomach tissue integrity. Molecular docking studies of compound **5k** into the catalytic binding pocket of COX-1 and COX-2 enzymes revealed a strong congruence with the obtained anti-inflammatory results. Moreover, the tested compounds possessed a reasonable drug-likeness with adequate physicochemical properties. Collectively, compound **5k** could be considered as a potential oral anti-inflammatory drug, which worth further examination and drug optimization.

## Figures and Tables

**Table 1:** In vitro inhibition of COX-1 and COX-2 enzymes by compounds **5d, 5j, 5k**, and **5m** compared to celecoxib using a fluorescent inhibitor screening assay kit.

Compound ID	IC_50_ μM ^a^ ± SEM		SI ^b^
	COX-1	COX-2	
**5d**	14.08 ± 0.47	0.49 ± 0.017	28.73
**5j**	6.403 ± 0.22	0.398 ± 0.014	16.09
**5k**	25.47 ± 0.86	0.266 ± 0.009	95.75
**5m**	57.31 ± 1.93	2.342 ± 0.08	24.47
**Celecoxib**	28.92 ± 0.93	0.293 ± 0.01	98.70
**Indomethacin**	0.0892 ± 0.003	0.202 ± 0.007	0.441

**^a^** Values represent IC_50_ (μM) and percent inhibition at saturation. **^b^** In vitro COX-2 selectivity index (COX-1 IC_50_/COX-2 IC_50_).

**Table 2:** Percentage of inhibition of carrageenan-induced paw edema in mice.

Compound ID	1 h	2 h	3 h
**5d**	3.43%	21.26%	27.32%
**5j**	28.35%	50.13%	62.41%
**5k**	31.46%	44.88%	54.89%
**5m**	3.43%	58.01%	39.85%
**Celecoxib**	0.31%	44.88%	56.14%
**Indomethacin**	15.89%	29.13%	32.33%

**Table 3:** Lipophilicity parameters of compounds **5d, 5j, 5k**, and **5m**.

Compound ID	iLOGP	XLOGP3	WLOGP	MLOGP	Silicos-IT LogP	Consensus Log P
**5d**	2.09	1.92	1.94	1.61	−0.70	1.37
**5j**	2.96	3.34	3.18	3.17	2.47	3.03
**5k**	3.52	3.84	3.95	3.83	2.43	3.51
**5m**	2.62	2.02	2.23	2.09	1.61	2.12

**Table 4:** Water solubility parameter of compounds **5d, 5j, 5k**, and **5m**.

Compound ID	ESOL Solubility (mg/mL)	ESOL Class	Ali Solubility (mg/mL)	Ali Class	Silicos-IT Solubility (mg/mL)	Silicos-IT Class
**5d**	9.59 × 10^−2^	Soluble	−2.33 × 10^−2^	Moderately soluble	2.63 × 10^−3^	Moderately Soluble
**5j**	8.22 × 10^−3^	Moderately Soluble	7.28 × 10^−5^	Moderately soluble	1.36 × 10^−5^	Poorly soluble
**5k**	3.34 × 10^−3^	Moderately Soluble	3.59 × 10^−3^	Moderately soluble	1.14 × 10^−5^	Poorly soluble
**5m**	7.29 × 10^−2^	Soluble	3.42 × 10^−4^	Moderately soluble	8.27 × 10^−4^	Moderately Soluble

**Table 5:** Pharmacokinetics of compounds **5d**, **5j**, **5k**, and **5m**.

Compound ID	GI Absorption	BBB Permanent	Pgp Substrate	CYP1A2 Inhibitor	CYP2C19 Inhibitor	CYP2C9 Inhibitor	CYP2D6 Inhibitor	CYP3A4 Inhibitor	Log Kp (cm/s)
**5d**	High	No	No	No	No	Yes	No	No	−7.32
**5j**	High	No	Yes	Yes	Yes	Yes	No	No	−6.34
**5k**	High	Yes	No	No	No	Yes	No	No	−6.14
**5m**	High	No	No	No	No	Yes	No	No	−7.31

**Table 6:** Drug likeness parameters of compounds **5d**, **5j**, **5k**, and **5m**.

Compound ID	Lipinski Violations	Ghose Violations	Veber Violations	Egan Violation	Muegge Violations	Bioavailability Score
**5d**	0	0	0	0	0	0.55
**5j**	0	0	0	0	0	0.55
**5k**	0	0	0	0	0	0.55
**5m**	0	0	0	0	0	0.55

## Data Availability

The original contributions presented in this study are included in the article/Supplementary Material. Further inquiries can be directed to the corresponding authors.

## Supplementary Materials

The following supporting information can be downloaded at https://www.mdpi.com/article/10.3390/ph18091326/s1.
