# Supplementary material for: Design, Synthesis, In Silico Docking, Multitarget Bioevaluation and Molecular Dynamic Simulation of Novel Pyrazolo[3,4-d]Pyrimidinone Derivatives as Potential In Vitro and In Vivo Anti-Inflammatory Agents
Source: Pharmaceuticals (Basel). 2025 Sep 4;18(9):1326. doi: 10.3390/ph18091326 (PMC12472512; doi:10.3390/ph18091326)
Supplement: Supplementary file 1 [file pharmaceuticals-18-01326-s001.zip › pharmaceuticals-3826339-supplementary.pdf]

Design, synthesis, *in-silico* docking, multitarget bio evaluation and molecular dynamic simulation of novel pyrazolo[3,4-*d*]pyrimidinone derivatives as potential in *vitro* and *in vivo* anti-inflammatory agents

Mostafa Roshdi[1,2], Mamdouh F. A. Mohamed[3,4,*], Eman A.M. Beshr[1,5], Hossameldin A. Aziz[6], Sahar M Gebril[7], Stefan Bräse[8,*], Aliaa M. Mohassab[1,5,*]

[1] *Department of Medicinal Chemistry, Faculty of Pharmacy, Minia University, Minia 61519, Egypt.*

[2] *Department of Medicinal Chemistry, Faculty of Pharmacy, Merit University, Sohag, Egypt.*

[3] *Department of Pharmaceutical Chemistry, Faculty of Pharmacy, Sohag University, Sohag 82524, Egypt.*

[4] *Department of Pharmaceutical Chemistry, Faculty of Pharmacy, New Valley UniversityThe institution will open in a new tab, 72511, New Valley, Egypt.*

[5] *Medicinal Chemistry Department, Faculty of Pharmacy, Minia National University, New Minia, Egypt.*

[6] *Department of Pharmaceutical Chemistry, Faculty of Pharmacy, New Valley University, New Valley 72511, Egypt.*

[7] *Department of Histology and Cell Biology, Faculty of Medicine, Sohag University, Sohag, Egypt.*
[8] *Institute for Biological and Chemical System, Karlsruhe Institute of Technology, 76131 Karlsruhe, Germany.*

***To whom correspondence should be addressed:**

Ass. Prof. Mamdouh F. A. Mohamed

Department of Pharmaceutical Chemistry, Faculty of Pharmacy, Sohag University, 82524- Sohag, Egypt.

E-mail address: mamdouhfawzy3@yahoo.com, mamdouh.fawzi@pharm.sohag.edu.eg

Prof. Dr. Stefan Bräse:

Institute for Biological and Chemical System, Karlsruhe Institute of Technology, 76131 Karlsruhe, Germany

E-mail address: *stefan.braese@kit.edu*

Dr. Aliaa M. Mohassab:

Department of Medicinal Chemistry, Faculty of Pharmacy, Minia University, Minia 61519, Egypt

E-mail address: alyaa.mohasab@mu.edu.eg

NMR spectral data

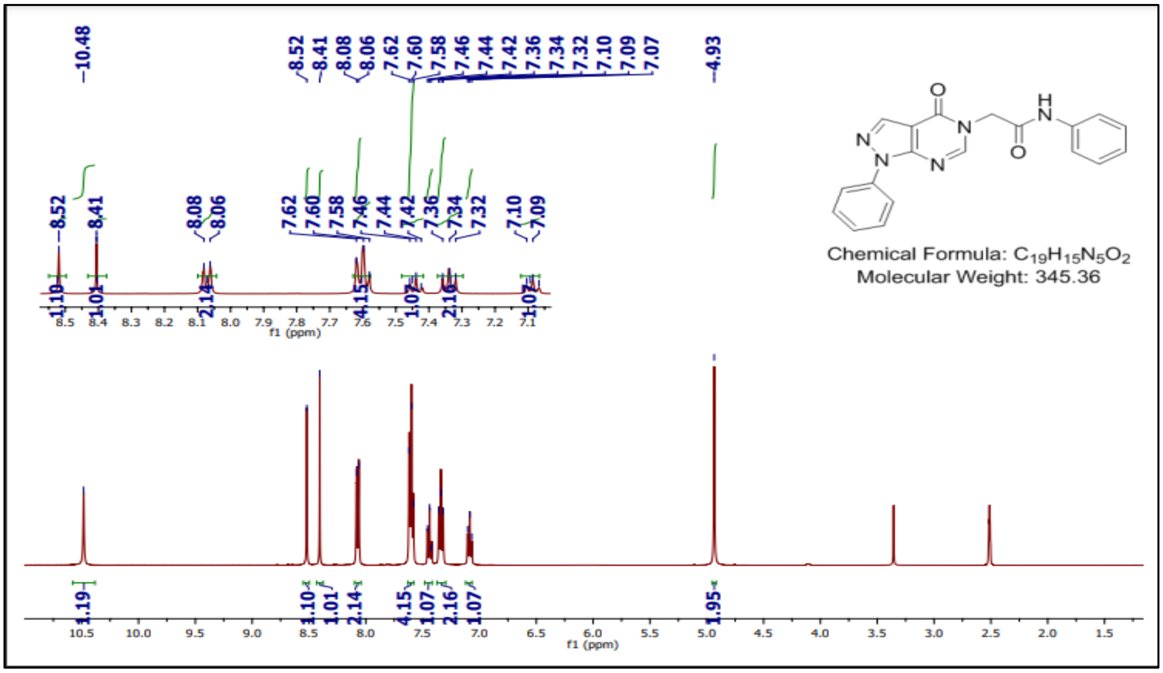

Figure S1. ^{1}H NMR for compound 5a

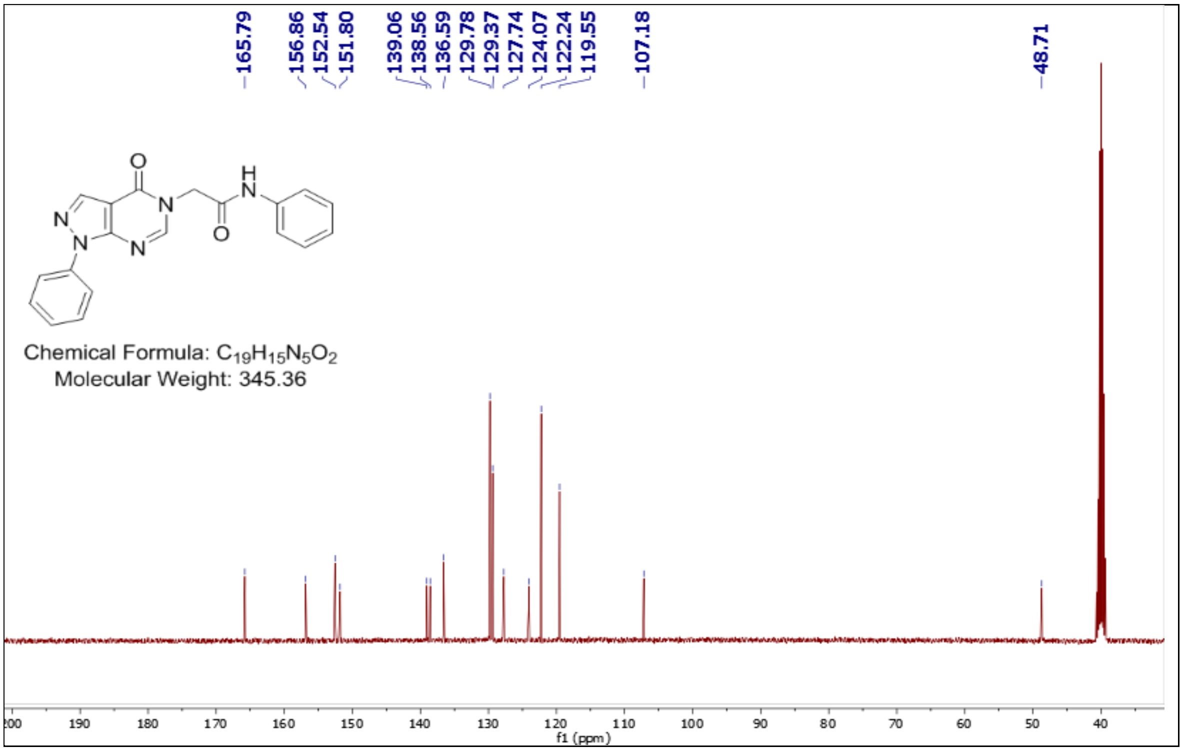

Figure S2. ^{13}C NMR for compound 5a

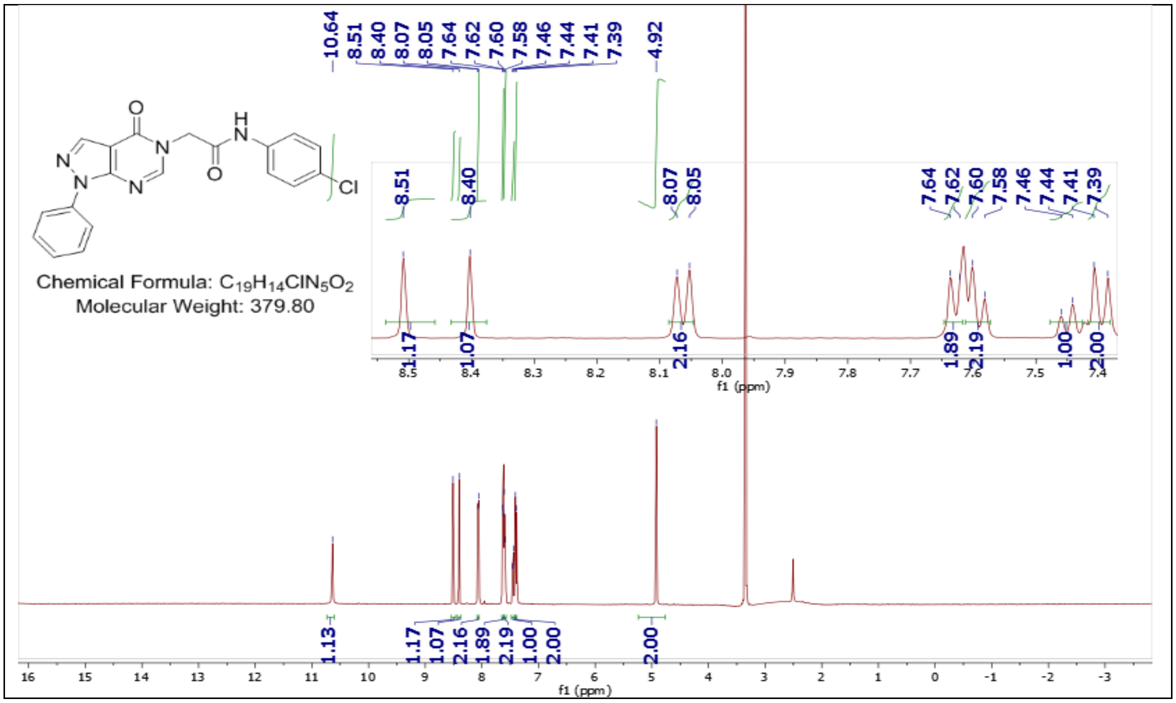

Figure S3.[1]**H NMR for compound 5b**

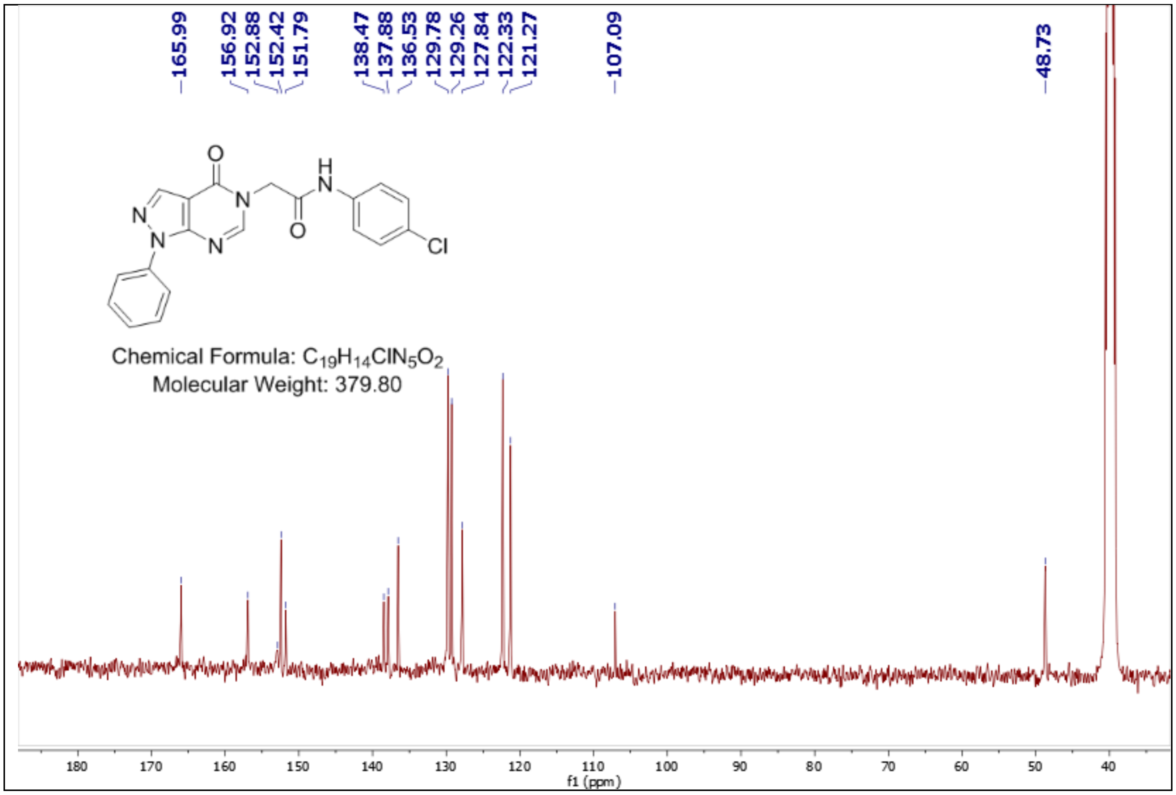

Figure S4.[13]**C NMR for compound 5b**

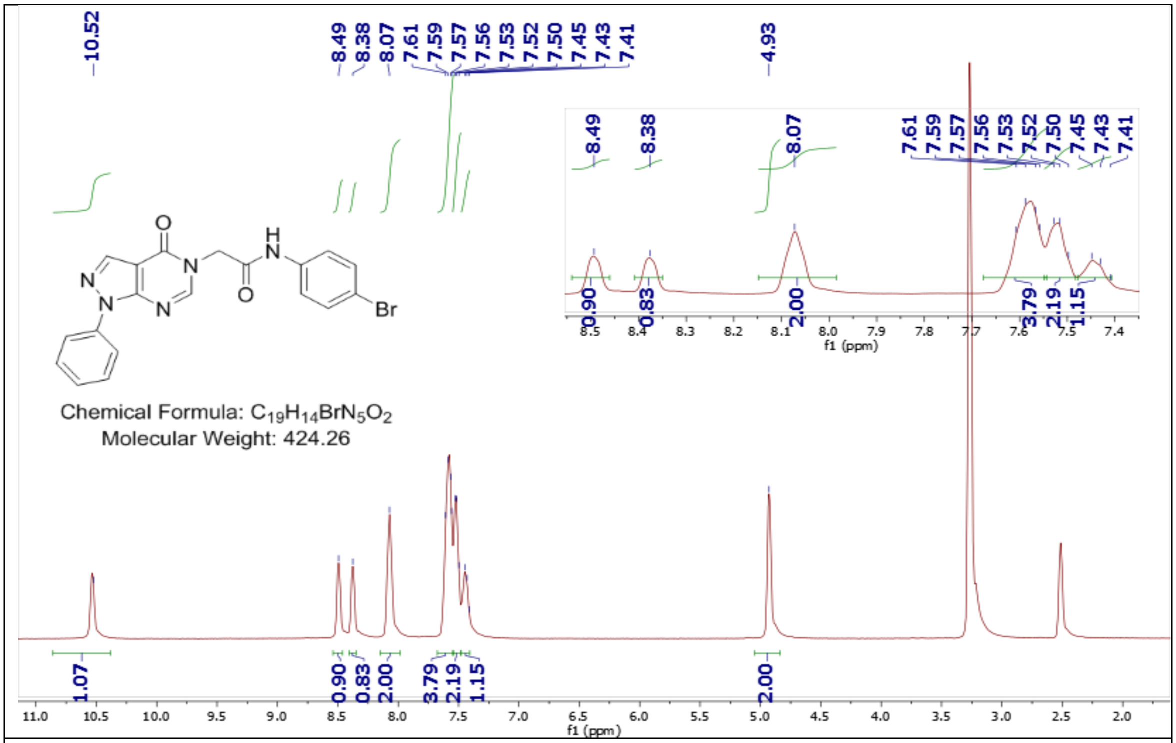

Figure S5. ^{1}H NMR for compound 5c

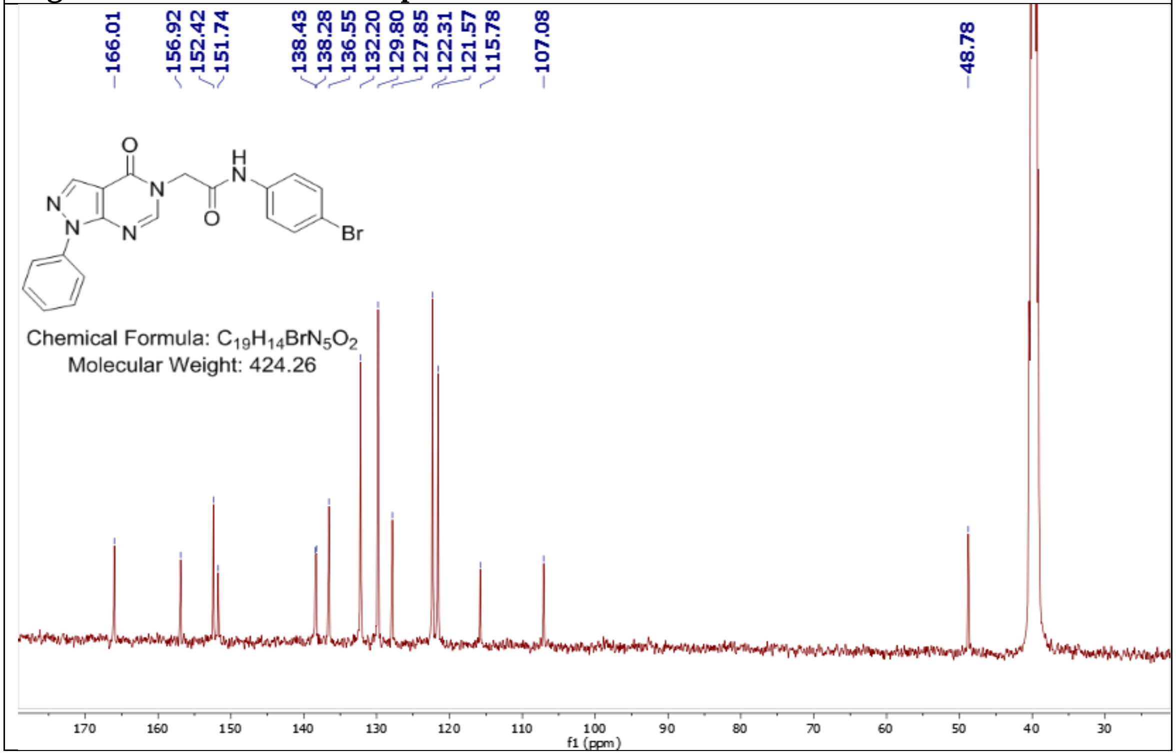

Figure S6. ^{13}C NMR for compound 5c

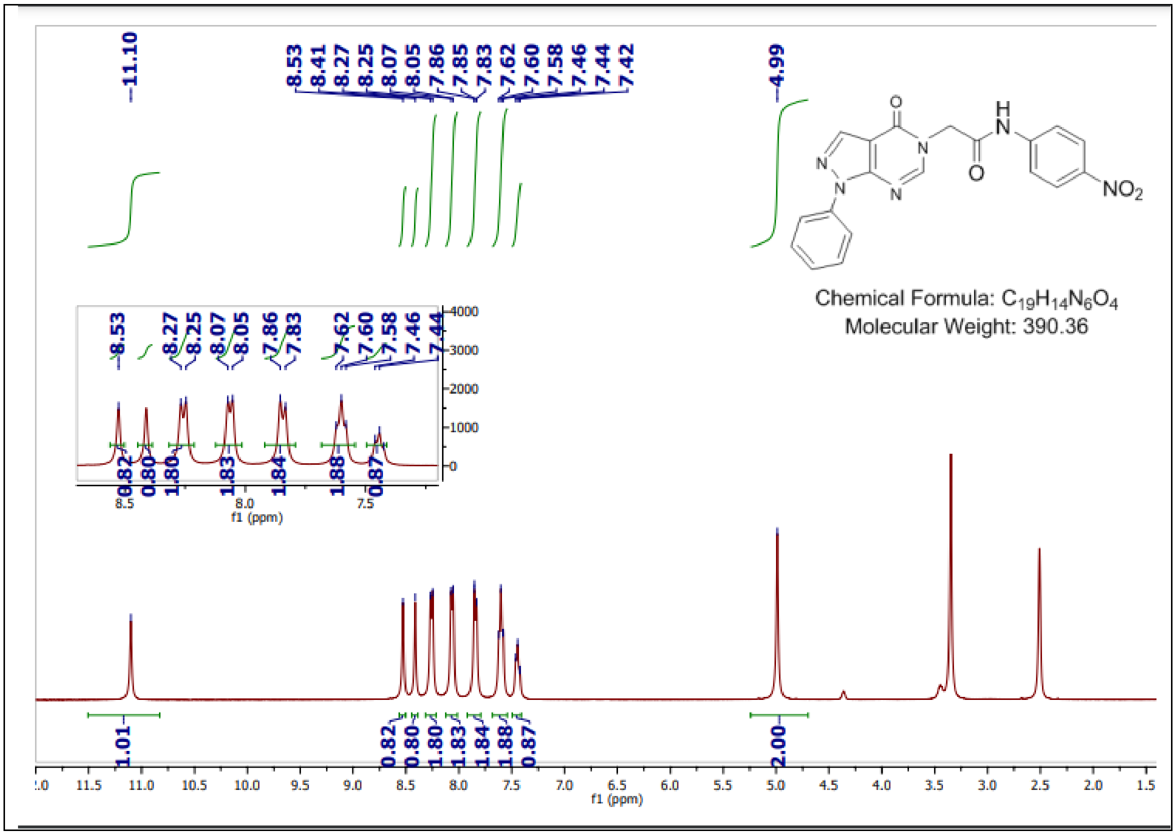

Figure S7. ^{1}H NMR for compound 5d

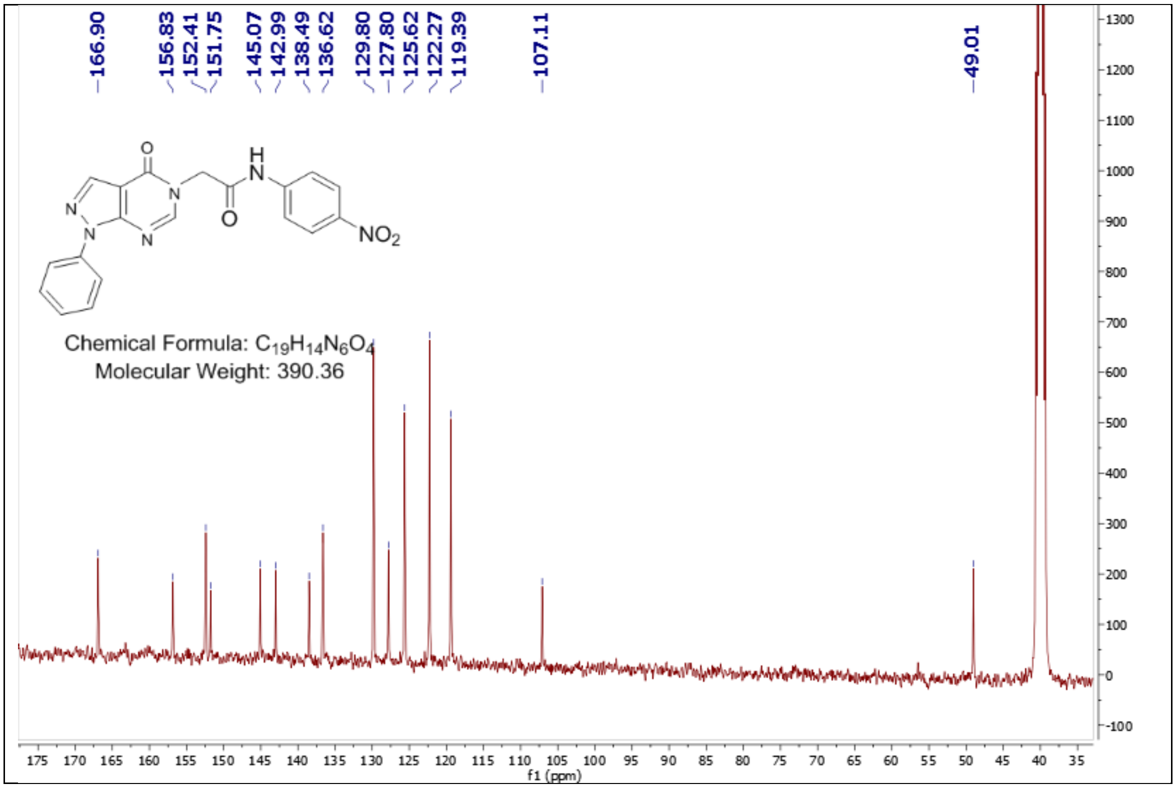

Figure S8. ^{13}C NMR for compound 5d

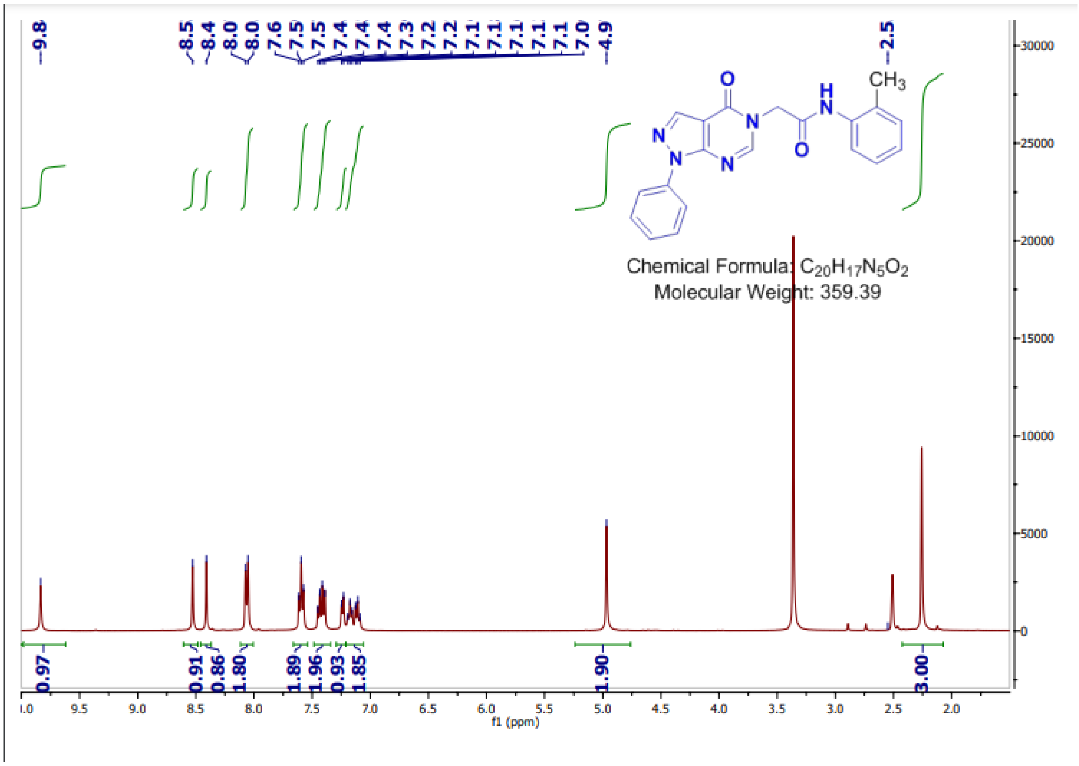

Figure S9. ^{1}HNMR for compound 5e

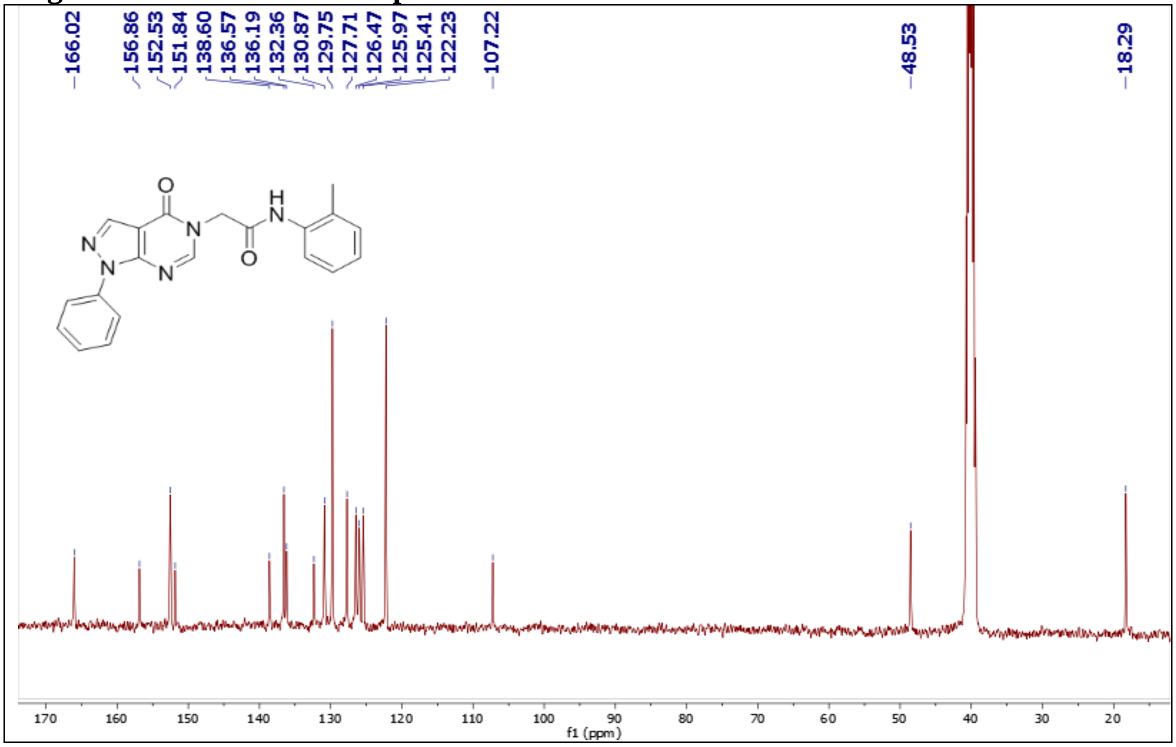

Figure S10. ^{13}C NMR for compound 5e

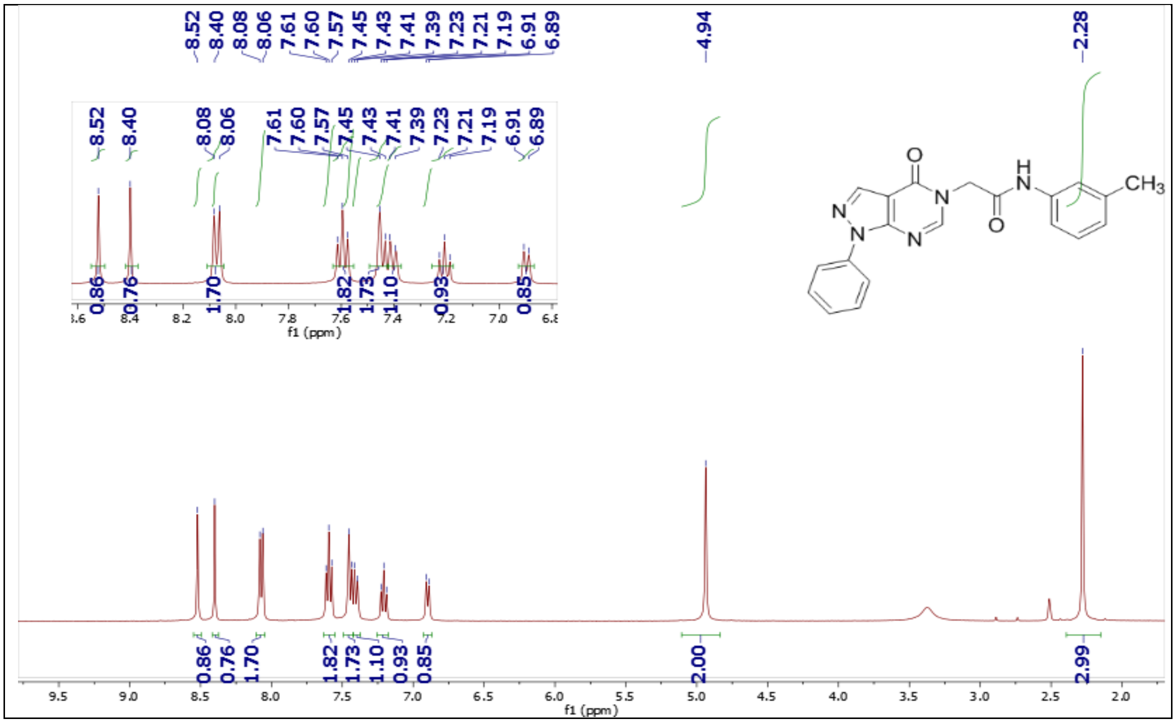

Figure S11. ^{1}H NMR for compound 5f

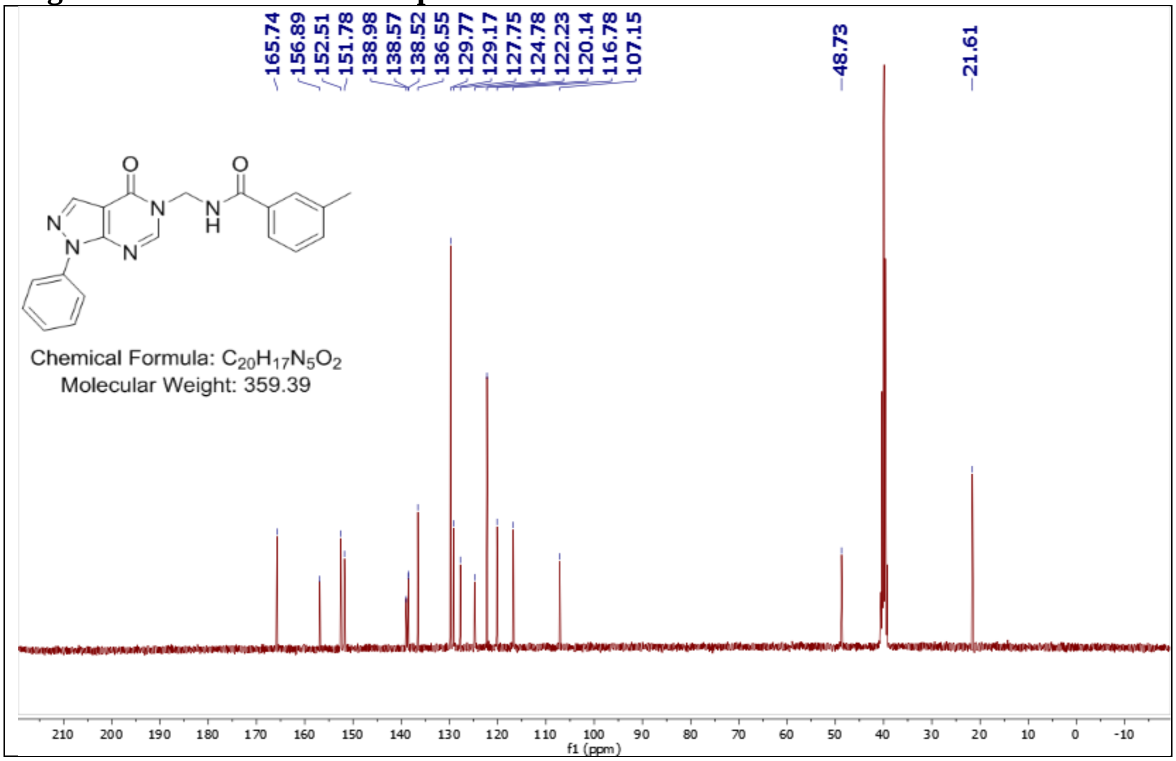

Figure S12. ^{13}C NMR for compound 5f

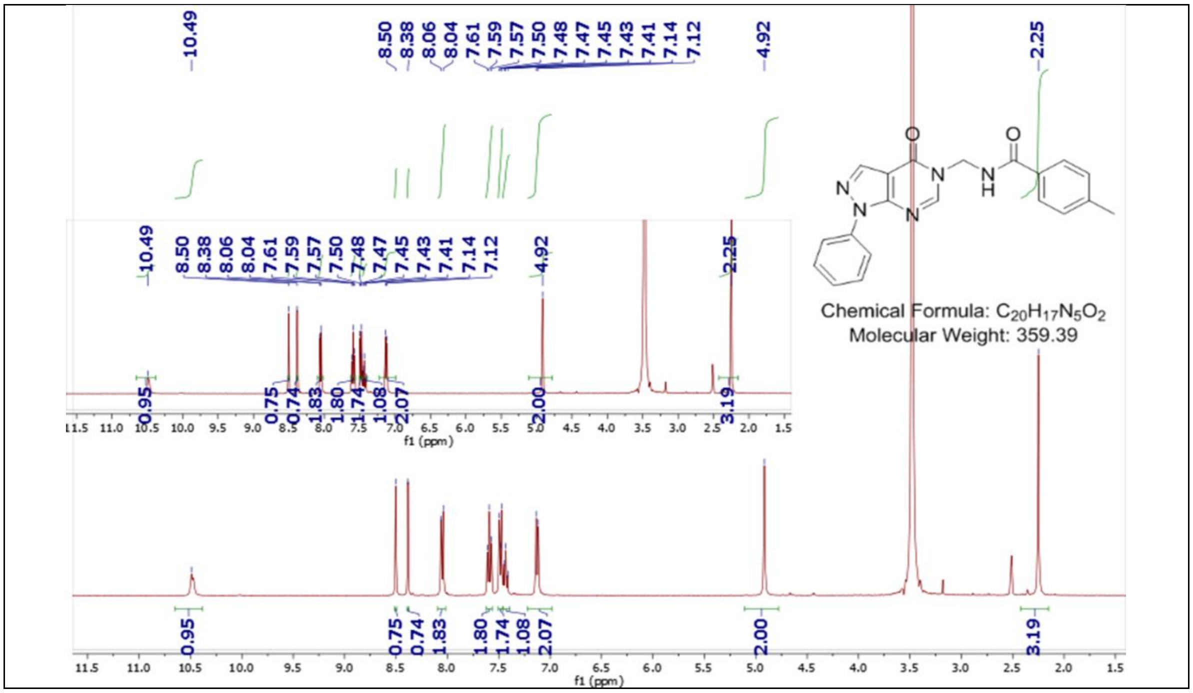

Figure S13. ^{1}H NMR for compound 5g

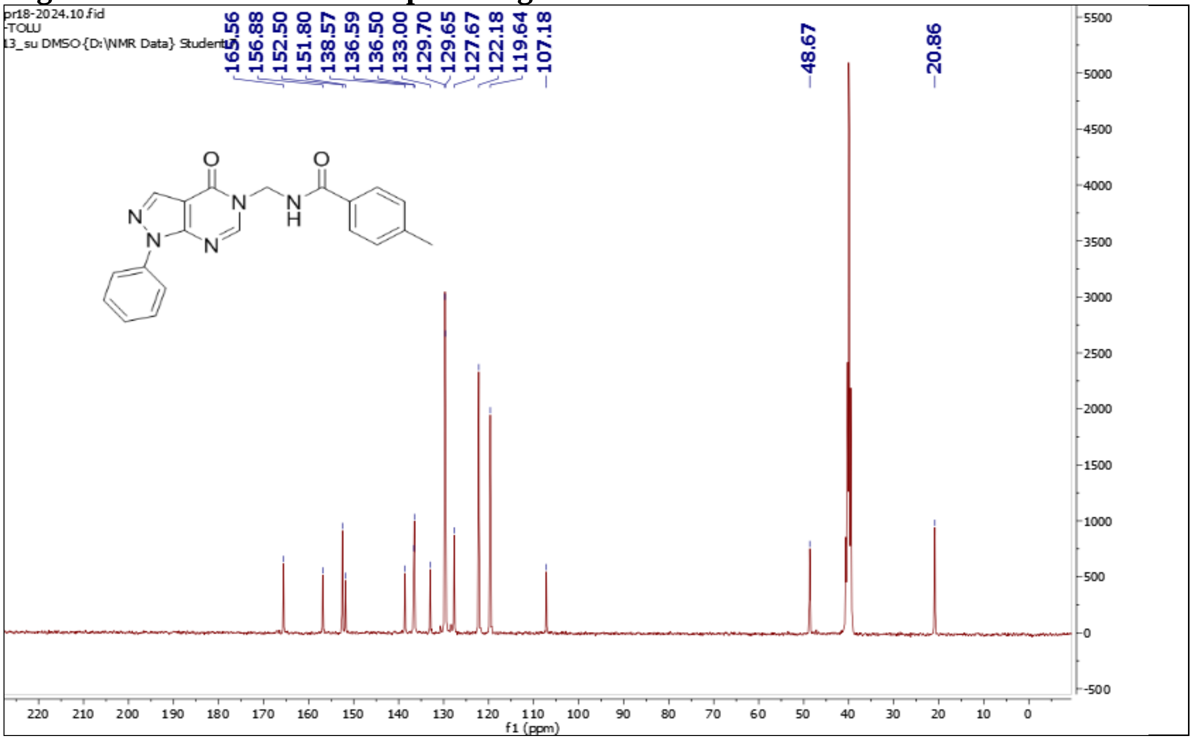

Figure S14. ^{13}C NMR for compound 5g

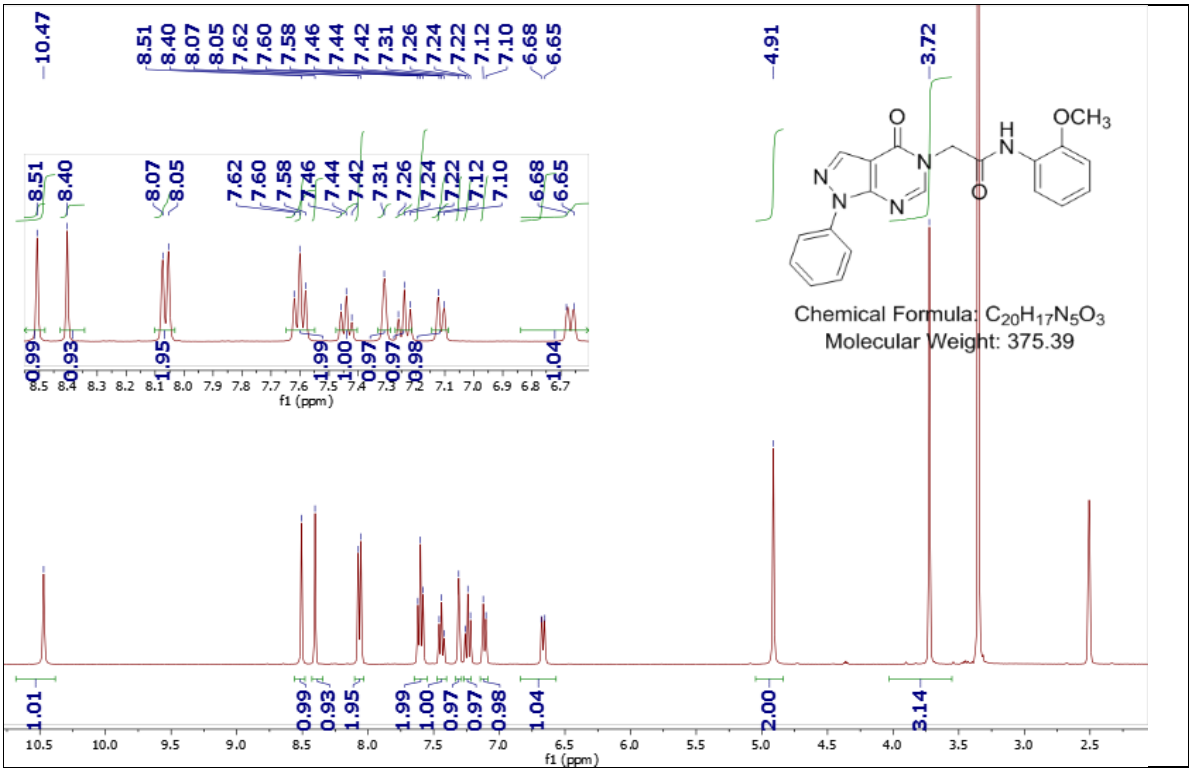

Figure S15. ^{1}H-NMR for compound 5h

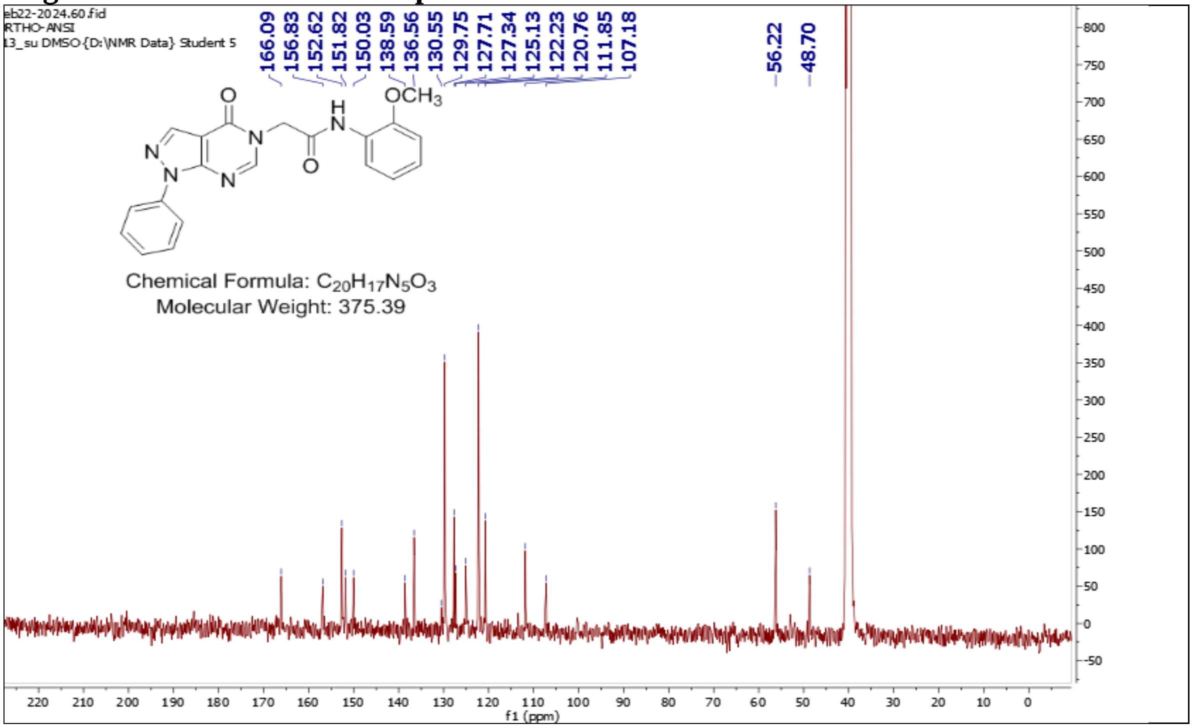

Figure S16. ^{13}CNMR for compound 5h

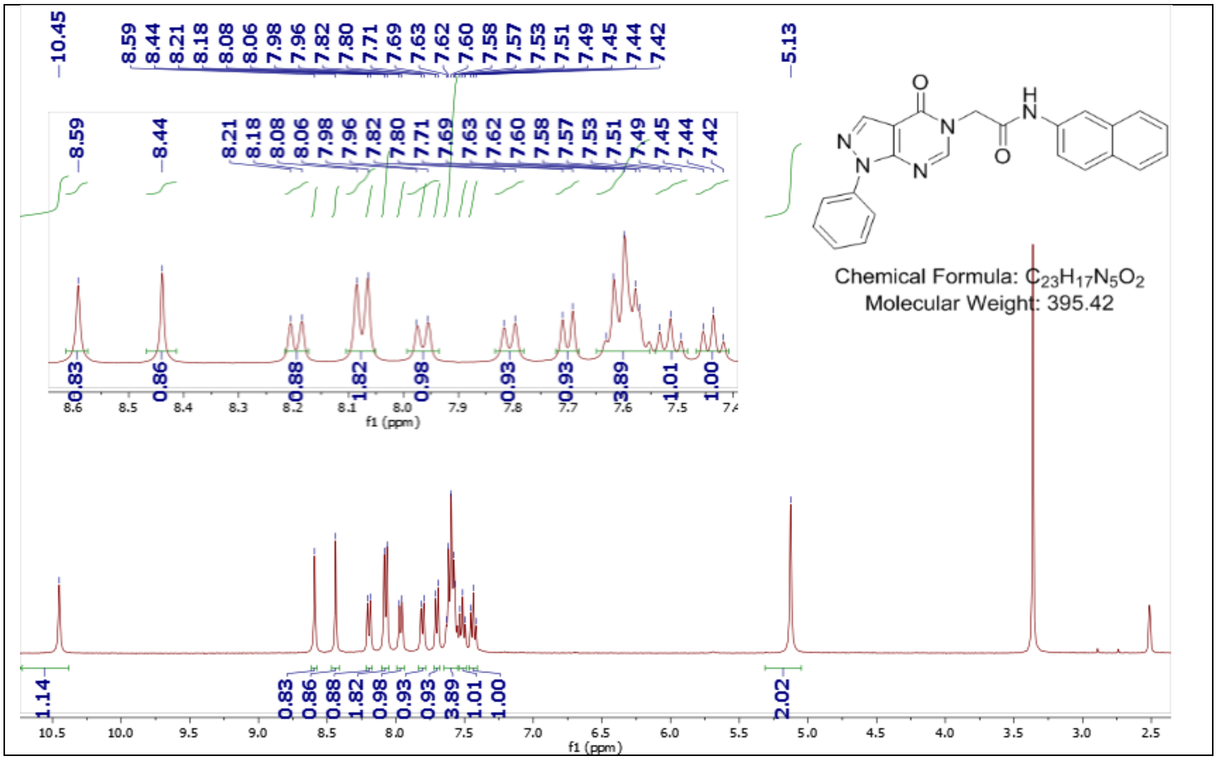

Figure S17. ^{1}H NMR for compound 5j

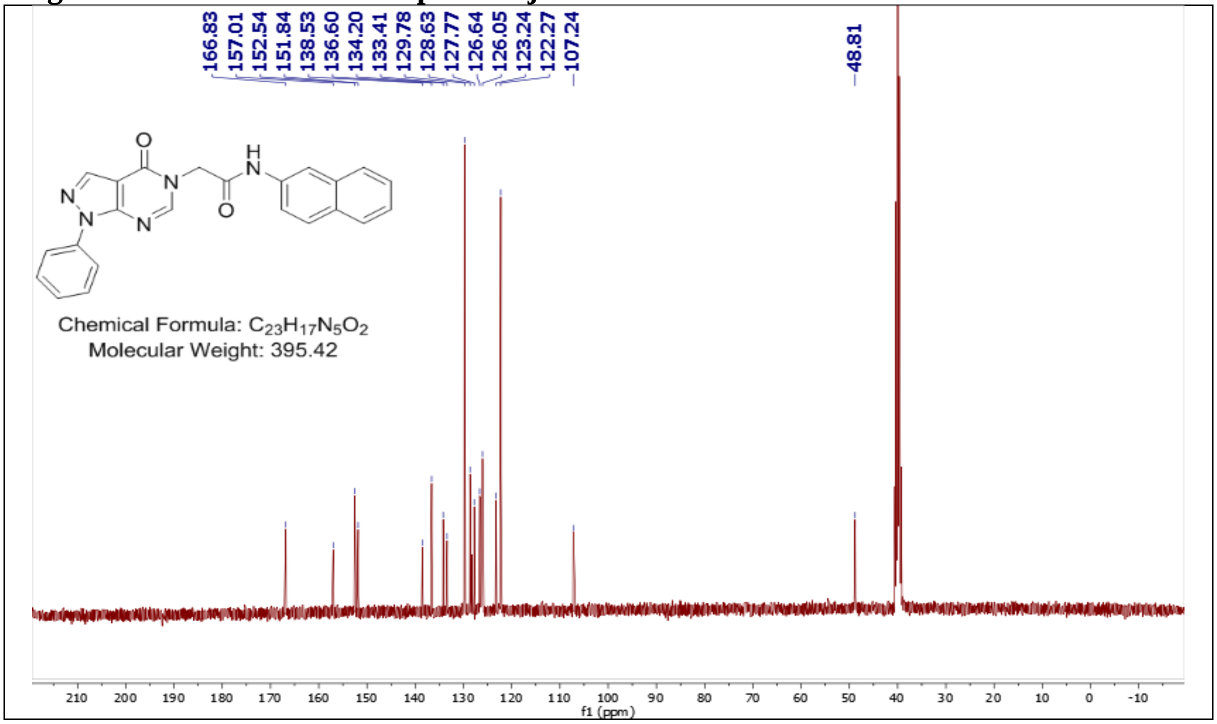

Figure S18. ^{13}C NMR for compound 5j

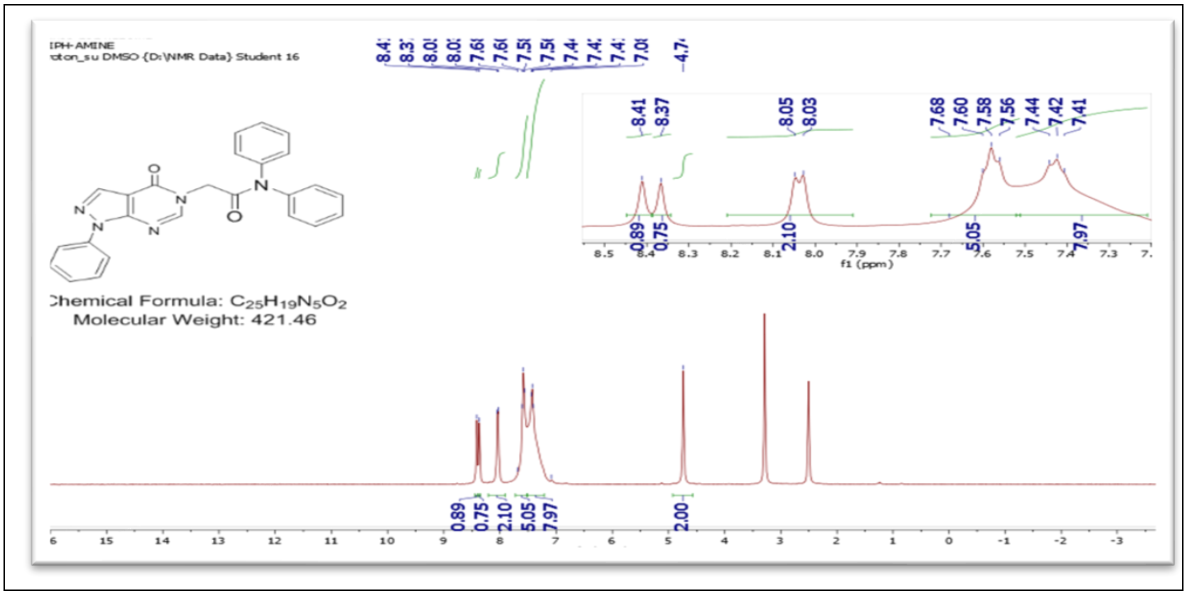

Figure S19. ^{1}H NMR for compound 5k

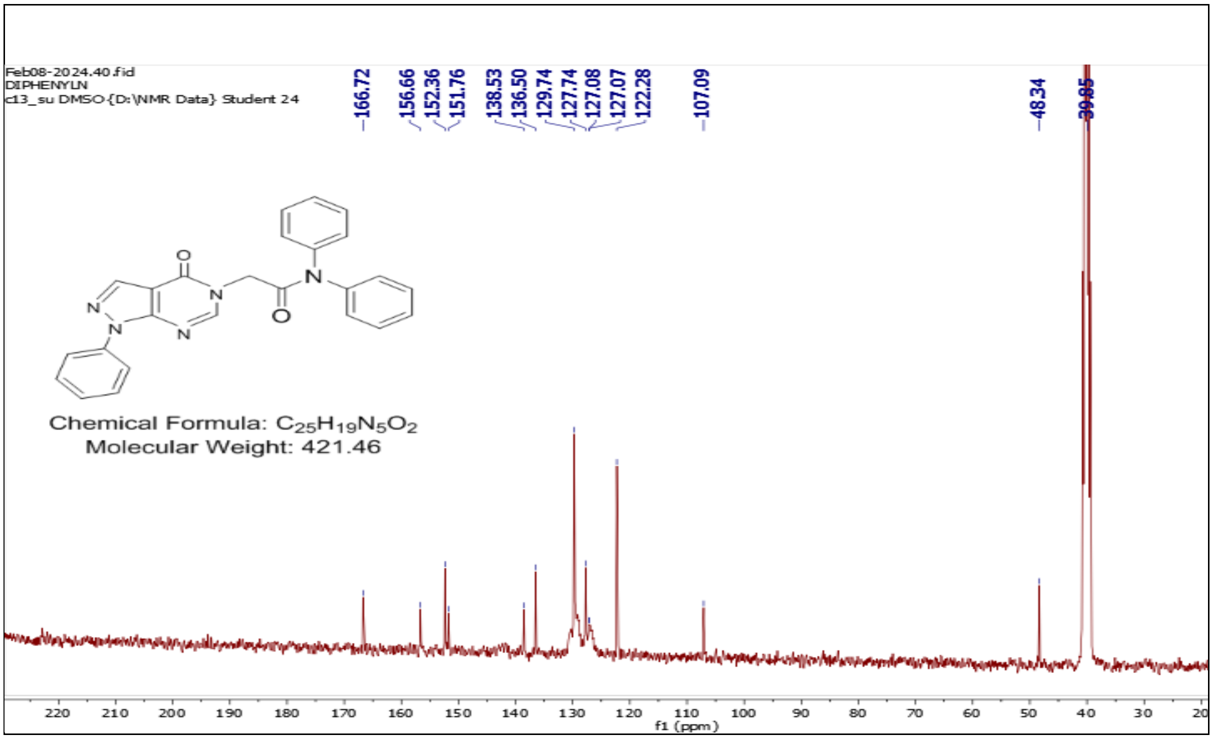

Figure S20. ^{13}C NMR for compound 5k

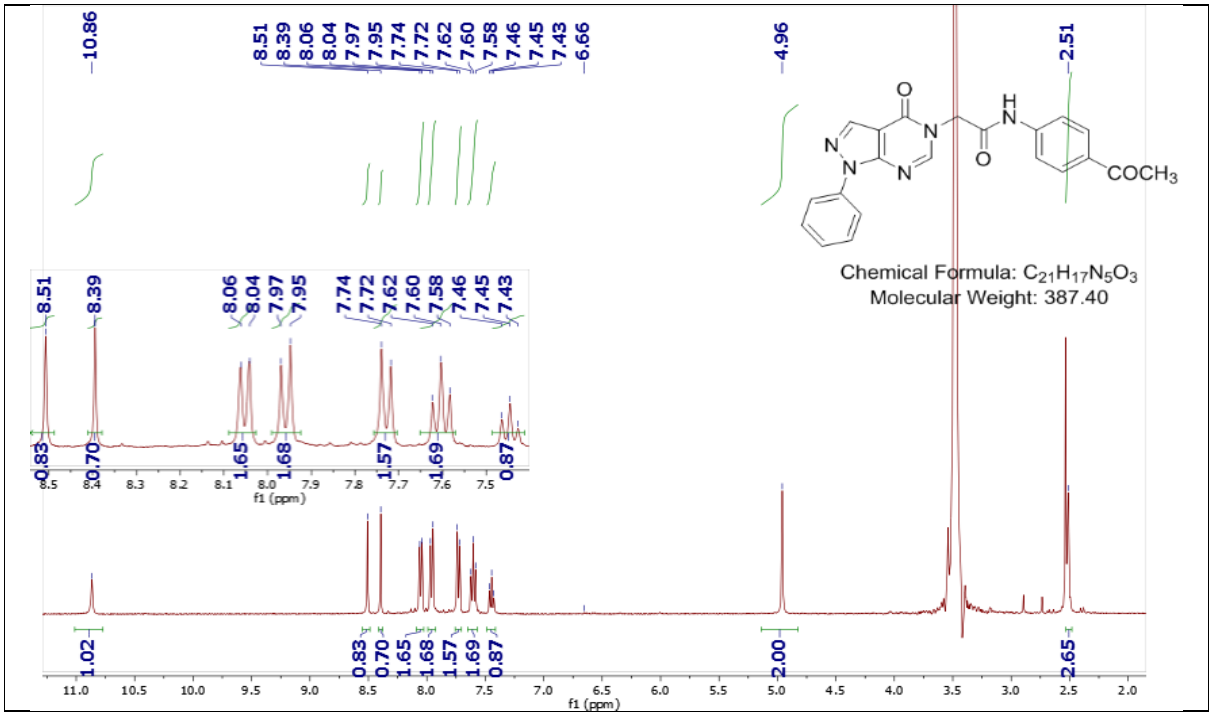

Figure S21. ^{1}H NMR for compound 5l

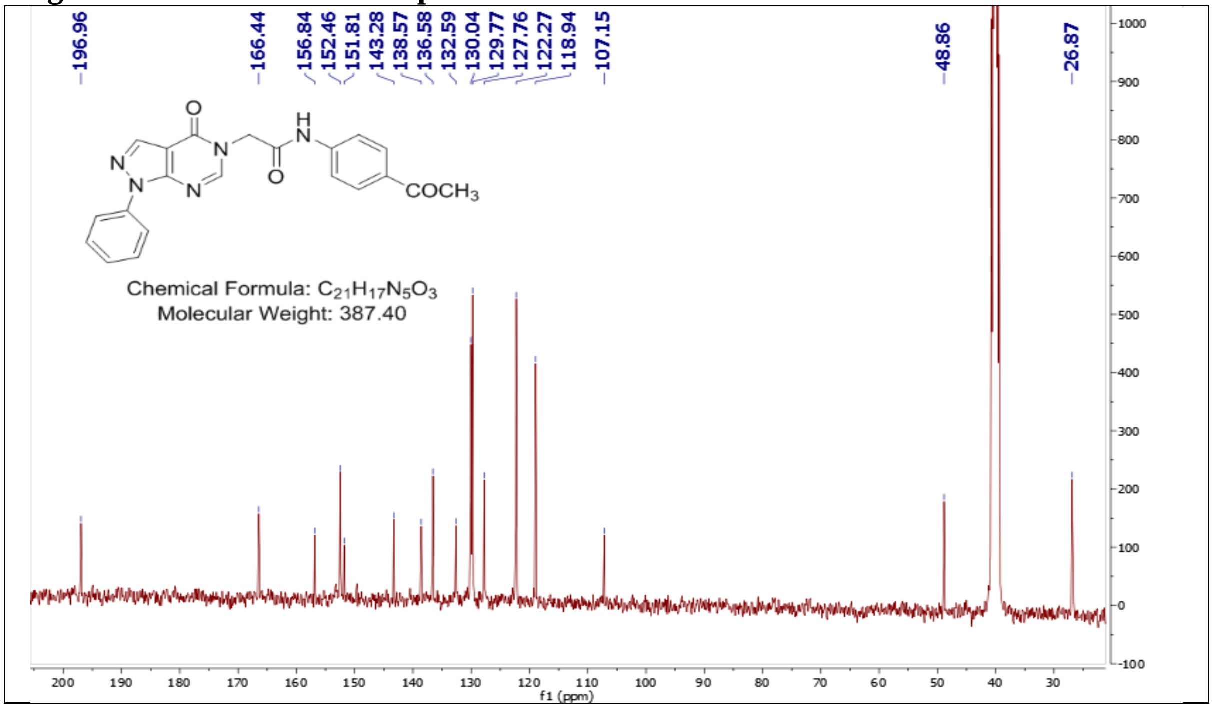

Figure S22. ^{13}C NMR for compound 5l

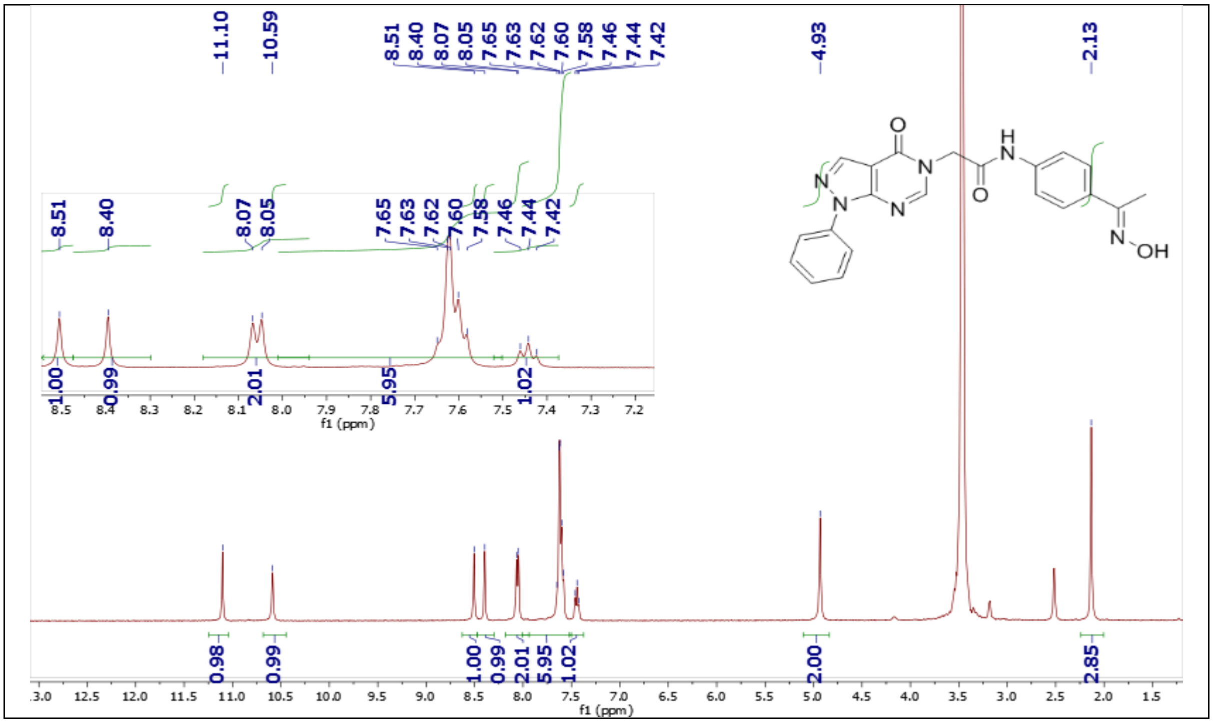

Figure S23. ^{1}H NMR for compound 5m

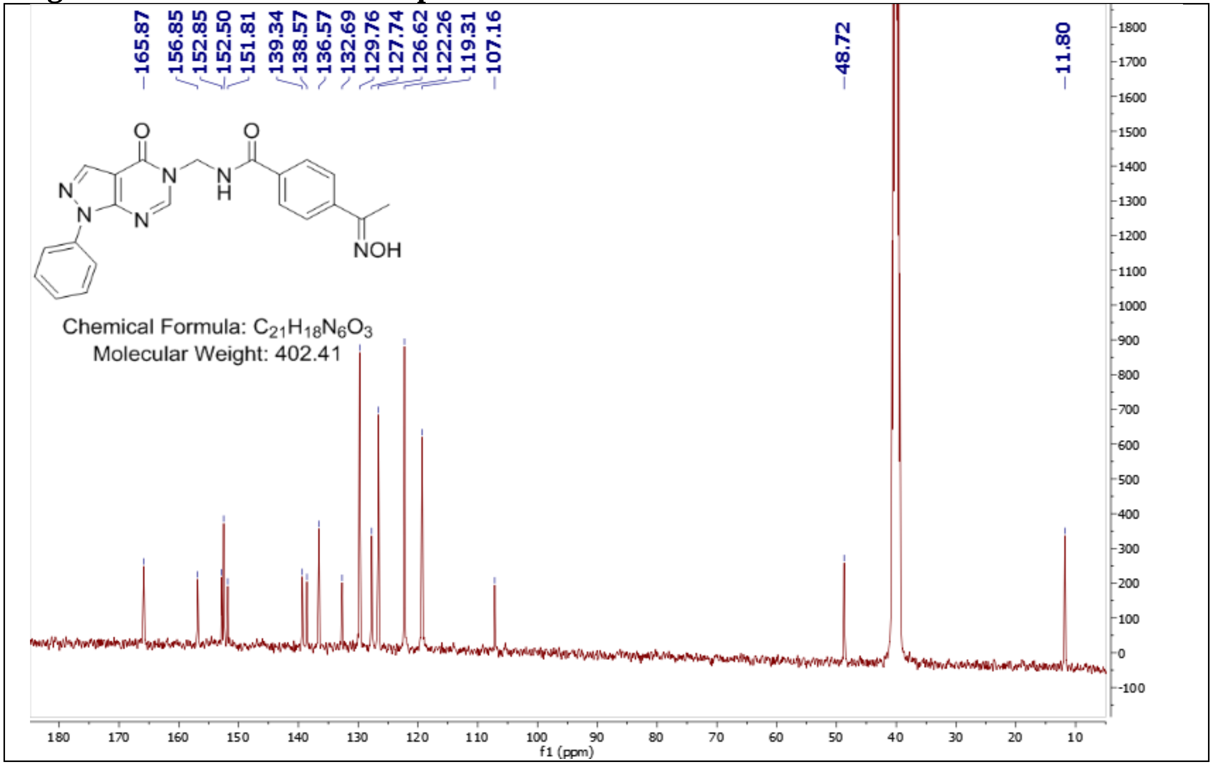

Figure S24. ^{13}C NMR for compound 5m

Figure S25-A. Elemental analysis data for the target compounds

جامعة الأزهر
Al-Azhar University
المركز الإقليمى للفطريات وتطبيقاتها
The Regional Center for Mycology and Biotechnology

Requester Data:

Name: **Dr. Mostafa Roshdy Koubissy**
Authority: Faculty of Pharmacy, Sohag University

Sample Data:

Thirteen samples had been submitted for elemental analysis.

Analysis Report:

Sample Code	C%	H%	N%
m. ansi	64.17	4.75	18.93
M. tolu	67.05	4.93	19.70
Naph	70.08	4.51	17.94
O. ansi	64.17	4.68	18.92
OCH_3. CYL	66.52	4.72	15.73
OCH_3. OXi	64.18	4.70	18.89
O. tolu	66.97	4.82	19.67
OXi. $COCH_3$	62.89	4.65	21.14
P. ansi	64.21	4.73	18.49
P. Br. ani	54.05	3.59	16.81
P. Cl.ani	60.23	3.64	18.69
P. NO_2. ani	58.62	3.71	21.80
P. tolu	67.06	4.93	19.65

INVESTIGATOR **DIRECTOR**

16-3-2024

تليفون: ٢٢٦٢٠٣٧٣ (٠٢٠٢) فاكس: ٢٢٦٢٠٣٧٣ (٠٢٠٢)
شارع المخيم الدائم - مدينة نصر - القاهرة
البريد الإلكتروني : rcmb@azhar.edu.eg
الموقع الإلكتروني:
http:// www.azhar.edu.eg.htm
http://www.azhar.edu.eg/pages/fungi_center.htm
Facebook: RCMB AZHAR
صندوق بريد ١١٧٥١ مدينة نصر القاهرة

Note: compounds M. tolu, Napth, O. O. tolu., OXi COCH3, P. ansi, P. Br.ani, P.Cl.ani, P.NO2.ani, P. tolu corresponding to compounds 5f, 5j, 5e, 5m, 5i, 5c, 5b, 5d and 5g, respectively.

Figure S25-B. Elemental analysis data for the target compounds

جامعة الأزهر
Al-Azhar University
المركز الإقليمي للفطريات وتطبيقاتها
The Regional Center for Mycology and Biotechnology

Requester Data:

Name: Dr. Mostafa Roshdy Koubissy
Authority: Faculty of Pharmacy, Sohag University

Sample Data:

Thirteen samples had been submitted for elemental analysis.

Analysis Report:

Sample Code	C%	H%	N%
Br. cyl	55.89	3.41	13.97
Br. oxi	54.01	3.40	16.75
CH_3 CYL	70.05	4.81	16.43
CH_3 oxi	67.02	4.89	19.67
Cl. CYL	62.78	3.77	15.58
Cl. Oxi	60.23	3.90	18.31
CO CH_3	65.29	4.53	18.30
Diphenamine	71.43	4.61	16.89
F.cyl	65.76	3.89	16.30
F. oxi	63.05	5.31	19.46
H. ani	66.29	4.42	20.57
H. cyl	68.89	4.40	17.12
H. oxi	66.31	4.49	20.47

INVESTIGATOR M. M

DIRECTOR M. Mansour 3.3.2021

جامعة الأزهر المركز الإقليمي للفطريات وتطبيقاتها

شارع المخيم الدائم - مدينة نصر - القاهرة
البريد الإلكتروني : remb@azhar.edu.eg
الموقع الإلكتروني:
صندوق بريد ١١٧٥١ مدينة نصر القاهرة
تليفون : (٠٢٠٢) ٢٢٦٢٠٣٧٣ فاكس : (٠٢٠٢) ٢٢٦٢٠٣٧٣
http:// www.azhar.edu.eg.htm
http://www.azhar.edu.eg/pages/fungi_center.htm
Facebook: RCMB AZHAR

Note: compounds Diphenylamine, H-ani, O-ansi, and $COCH_3$ are corresponding to compounds 5k, 5a, 5h and 5l, respectively

Figure S26. *In vitro* inhibition of COX-1 and COX-2 enzymes by compounds **5d, 5j, 5k**, and **5m** compared to celecoxib using a fluorescent inhibitor screening assay kit.

ser	Compound			COX1/2		SI
	code	MW	conc. uM	IC50 ug/ml		
				COX1	COX2	
**1**	**(5J)**	---	---	**6.403**±0.22	**0.398**±0.014	**16.09**
**2**	**(5k)**	---	---	**25.47**±0.86	**0.266**±0.009	**95.75**
**3**	**(5m)**	---	---	**57.31**±1.93	**2.342**±0.08	**24.47**
**4**	**(5d)**	---	---	**14.08**±0.47	**0.49**±0.017	**28.73**
*	**Indomethacin**	---	---	**0.089±0.003**	**0.202**±0.007	**0.441**
**	**Celecoxib**	---	---	**28.92**±0.93	**0.293**±0.01	**98.7**

Detailed result

**COX1**		[redacted]										
Code	IC50	Conc	log	%inh	T2	T1	ΔT	RFU2	RFU1	ΔRFU	slope	K.Activity
**5j**		100	2	80.1	5	0	30	22563	0	22563	22650	3.9846
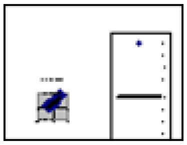												
		10	1	57.9	5	0	30	47691	0	47691	22650	8.4223
		1	0	19.2	5	0	30	91532	0	91532	22650	16.165
		0.1	-1	7.85	5	0	30	104361	0	104361	22650	18.43
		0.01	-2	1.32	5	0	30	111754	0	111754	22650	19.736
EC				0	5	0	30	113251	0	113251	22650	20
Code	IC50	conc	log	%inh	T2	T1	ΔT	RFU2	RFU1	ΔRFU	slope	K.Activity
**5k**		100	2	74.9	5	0	30	28433	0	28433	22650	5.0213
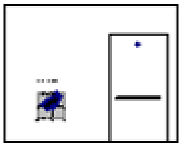												
		10	1	31.4	5	0	30	77641	0	77641	22650	13.711
		1	0	15	5	0	30	96246	0	96246	22650	16.997
		0.1	-1	5.18	5	0	30	107385	0	107385	22650	18.964
		0.01	-2	0.72	5	0	30	112431	0	112431	22650	19.855

Code	IC50	conc	log	%inh	T2	T1	ΔT	RFU2	RFU1	ΔRFU	slope	K.Activity
EC				0	5	0	30	113251	0	113251	22650	20

Code	IC50	conc	log	%inh	T2	T1	ΔT	RFU2	RFU1	ΔRFU	slope	K.Activity
**5m**		100	2	69.2	5	0	30	34891	0	34891	22650	6.1618
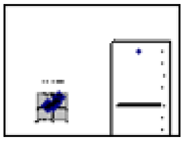		10	1	24.6	5	0	30	85391	0	85391	22650	15.08
		1	0	12.3	5	0	30	99267	0	99267	22650	17.531
		0.1	-1	6.51	5	0	30	105876	0	105876	22650	18.698
		0.01	-2	0.27	5	0	30	112942	0	112942	22650	19.946
EC				0	5	0	30	113251	0	113251	22650	20

Code	IC50	Conc	log	%inh	T2	T1	ΔT	RFU2	RFU1	ΔRFU	slope	K.Activity
**5d**		100	2	76	5	0	30	27154	0	27154	22650	4.7954
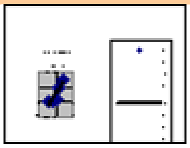		10	1	49.5	5	0	30	57239	0	57239	22650	10.108
		1	0	8.07	5	0	30	104113	0	104113	22650	18.386
		0.1	-1	1.77	5	0	30	111247	0	111247	22650	19.646
		0.01	-2	0.25	5	0	30	112969	0	112969	22650	19.95
EC				0	5	0	30	113251	0	113251	22650	20

Code	IC50	Conc	log	%inh	T2	T1	ΔT	RFU2	RFU1	ΔRFU	slope	K.Activity
**Celecoxib**		100	2	75.8	5	0	30	27428	0	27428	22650	4.8438
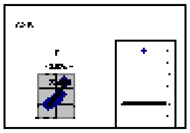		10	1	31	5	0	30	78174	0	78174	22650	13.806
		1	0	9.6	5	0	30	102382	0	102382	22650	18.081
		0.1	-1	1.65	5	0	30	111381	0	111381	22650	19.67
		0.01	-2	0.34	5	0	30	112865	0	112865	22650	19.932
EC				0	5	0	30	113251	0	113251	22650	20

5j

$y = 20.755x + 33.263$

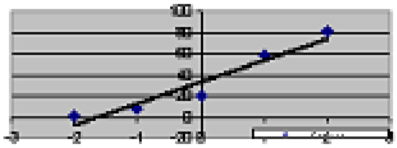

5k

$y = 17.46x + 25.451$

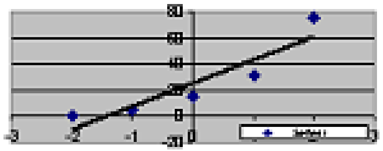

5m

$y = 15.593x + 22.584$

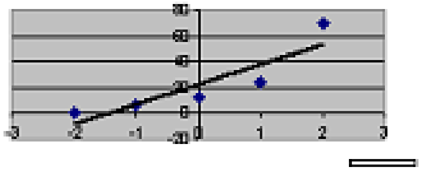

Celecoxib

$y = 18.02x + 23.668$

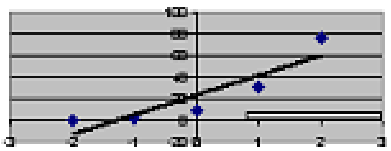

5d

$y = 19.924x + 27.113$

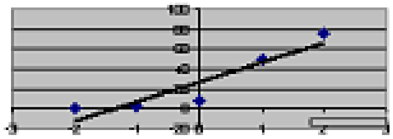

COX2

Code	IC50	conc	log	%inh	T2	T1	ΔT	RFU2	RFU1	ΔRFU	slope	K.Activity
**5j**		100	2	54.8	5	0	30	6537	0	6537	2892	9.0415
		10	1	9.91	5	0	30	13027	0	13027	2892	18.018
		1	0	-103	5	0	30	29423	0	29423	2892	40.696
		0.1	-1	-285	5	0	30	55735	0	55735	2892	77.089
		0.01	-2	-425	5	0	30	75922	0	75922	2892	105.01
EC				0	5	0	30	86773	0	86773	17355	20
	[redacted]		[redacted]		[redacted]		[redacted]		[redacted]			[redacted]

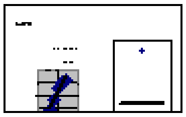

Code	IC50	conc	log	%inh	T2	T1	ΔT	RFU2	RFU1	ΔRFU	slope	K.Activity
**5k**		100	2	67.8	5	0	30	4659	0	4659	2892	6.444
		10	1	14.6	5	0	30	12342	0	12342	2892	17.071
		1	0	-76	5	0	30	25446	0	25446	2892	35.195
		0.1	-1	-266	5	0	30	52939	0	52939	2892	73.221
		0.01	-2	-394	5	0	30	71427	0	71427	2892	98.793
EC				0	5	0	30	86773	0	86773	17355	20
	[redacted]		[redacted]		[redacted]		[redacted]		[redacted]			[redacted]

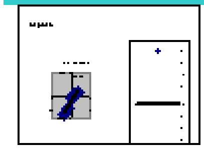

Code	IC50	conc	log	%inh	T2	T1	ΔT	RFU2	RFU1	ΔRFU	slope	K.Activity
**5m**		100	2	46.6	5	0	30	7728	0	7728	2892	10.689
		10	1	-103	5	0	30	29419	0	29419	2892	40.69
		1	0	-273	5	0	30	53884	0	53884	2892	74.528
		0.1	-1	-448	5	0	30	79238	0	79238	2892	109.6
		0.01	-2	-481	5	0	30	84036	0	84036	2892	116.23
	[redacted]		[redacted]		[redacted]		[redacted]		[redacted]			[redacted]

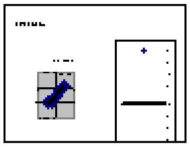

Code	IC50	conc	log	%inh	T2	T1	ΔT	RFU2	RFU1	ΔRFU	slope	K.Activity
**5d**		100	2	76.6	5	0	30	3381	0	3381	2892	4.6763
		10	1	-15	5	0	30	16592	0	16592	2892	22.949
		1	0	-167	5	0	30	38633	0	38633	2892	53.434
		0.1	-1	-301	5	0	30	57929	0	57929	2892	80.123

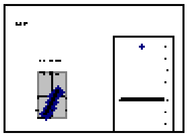

Code	IC50	conc	log	%inh	T2	T1	ΔT	RFU2	RFU1	ΔRFU	slope	K.Activity
		0.01	-2	-402	5	0	30	72546	0	72546	2892	100.34
EC				0	5	0	30	86773	0	86773	17355	20
	[redacted]	[redacted]	[redacted]		[redacted]		[redacted]		[redacted]	[redacted]	[redacted]	[redacted]
Code	IC50	conc	log	%inh	T2	T1	ΔT	RFU2	RFU1	ΔRFU	slope	K.Activity
**Celecoxib**		100	2	80	5	0	30	2896	0	2896	2892	4.0055
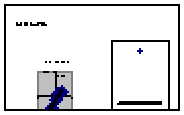		10	1	6.36	5	0	30	13541	0	13541	2892	18.729
		1	0	-106	5	0	30	29772	0	29772	2892	41.178
		0.1	-1	-275	5	0	30	54288	0	54288	2892	75.087
		0.01	-2	-383	5	0	30	69861	0	69861	2892	96.627
EC				0	5	0	30	86773	0	86773	17355	20
	[redacted]		[redacted]		[redacted]		[redacted]		[redacted]	[redacted]		[redacted]

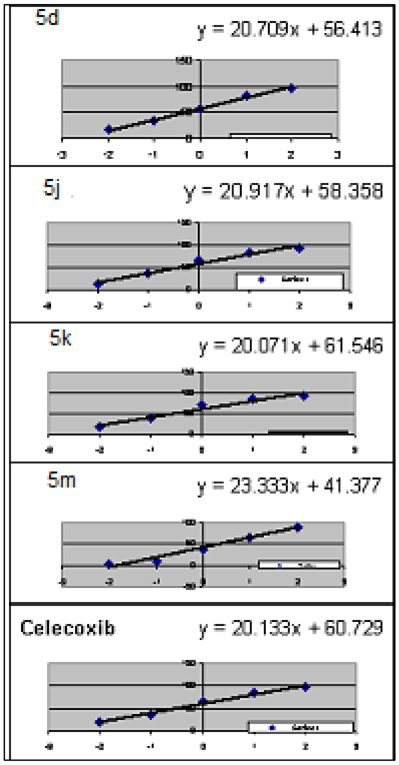

Laboratory Protocol: Estimation of COX-1 and COX-2 IC50 Values

1. Principle

This protocol utilizes a colorimetric assay to measure the peroxidase activity of Prostaglandin G/H Synthase (COX enzyme). The COX enzyme converts Arachidonic Acid (AA) to Prostaglandin G2 (PGG2), which is then reduced to Prostaglandin H2 (PGH2) by the inherent peroxidase activity. This peroxidase reaction co-oxidizes a chromogen, such as N,N,N',N'-Tetramethyl-p-phenylenediamine (TMPD), resulting in a colored product that can be measured spectrophotometrically at 590-630 nm. The inhibition of COX enzyme by a test compound reduces the formation of this colored product, allowing for the calculation of percentage inhibition and subsequent IC50 values.

2. Materials and Reagents

- **Enzymes:**
  - Ovine COX-1 (purified, commercially available)
  - Human recombinant COX-2 (commercially available)
- **Reference Compounds:**
  - Indomethacin (COX-1/2 reference inhibitor)
  - Celecoxib (selective COX-2 reference inhibitor)
- **Test Compound(s):** Your compound(s) of interest.
- **Substrate:** Arachidonic Acid (AA). Prepare a stock solution in ethanol. **Note:** AA is unstable; aliquot and store under inert gas (e.g., Argon) at -80°C.
- **Cofactor:** Hemin. Prepare a stock solution in DMSO.
- **Chromogen:** N,N,N',N'-Tetramethyl-p-phenylenediamine (TMPD) dihydrochloride. Prepare fresh in assay buffer.
- **Assay Buffer:** 100 mM Tris-HCl buffer, pH 8.0. May contain 1-5 mM EDTA and 1-2 µM Phenol (acts as a cofactor for COX-2).
- **Solvents:** Dimethyl Sulfoxide (DMSO), Ethanol (absolute). Ensure DMSO concentration is consistent across all wells (typically ≤1% final concentration).
- **Equipment:**
  - 96-well flat-bottom microplate (clear for reading)
  - Multichannel pipettes and reagent reservoirs
  - Microplate reader capable of reading absorbance at 590-630 nm (kinetic mode preferred)
  - Plate shaker (optional, for mixing)
  - Water bath or incubator (set to 37°C)

3. Safety Precautions

- Wear appropriate PPE: lab coat, gloves, and safety glasses.
- Arachidonic Acid is an irritant. Handle with care.

- DMSO is a known solvent that can facilitate the transport of chemicals through the skin. Handle all compound/DMSO solutions with extra caution.
- Follow all waste disposal regulations for chemical and biological waste.

4. Procedure

4.1. Preparation of Solutions

1. **Compound Dilutions:**
    - Prepare 10 mM stock solutions of Celecoxib, Indomethacin, and your test compound(s) in 100% DMSO.
    - Using the assay buffer, perform a serial dilution (e.g., 1:5 or 1:10) to create a range of concentrations (typically 5 concentrations) for each compound. The final top concentration in the assay will be 10-100 µM, depending on expected potency. **Include a DMSO-only control (0% inhibition) and a vehicle control (100% inhibition, e.g., high dose of Indomethacin).**
    - Keep all dilutions on ice.
2. **Enzyme Solutions:**
    - **COX-1 Working Solution:** Dilute ovine COX-1 in ice-cold assay buffer to a final concentration of ~50-100 U/mL. Add Hemin to a final concentration of 1 µM.
    - **COX-2 Working Solution:** Dilute human recombinant COX-2 in ice-cold assay buffer to a final concentration of ~50-100 U/mL. Add Hemin to a final concentration of 1 µM.
    - *Note: The exact enzyme units (U) required must be determined by a preliminary activity test to ensure the reaction is linear and within the dynamic range of the assay.*
3. **Substrate/Chromogen Solution (Prepare Fresh):**
    - To a tube containing assay buffer, add TMPD to a final concentration of 0.2-0.5 mM.
    - Add Arachidonic Acid to a final concentration of 100 µM. Vortex gently.
    - Keep this solution protected from light and on ice until use.

4.2. Assay Setup (Performed in duplicate or triplicate)
This is a typical protocol for a final reaction volume of 200 µL/well.

Step	Action	Vol/Well	Component	Final Concentration/Note
**1**	Pre-incubate compound with enzyme	160 µL	140 µL Enzyme (COX-1 or COX-2) + 20 µL Compound Dilution	[Compound] is 10x final desired conc.
**2**	Incubation	-	Incubate plate for 10-15 min at 25°C (room temp)	Allows inhibitor-enzyme binding
**3**	Initiate Reaction	80 µL	Add Substrate/Chromogen Solution (AA + TMPD)	Final [AA] = 20 µM, [TMPD] = ~0.1 mM

Step	Action	Vol/Well	Component	Final Concentration/Note
**4**	Read Plate	-	Immediately transfer to plate reader and measure kinetic absorbance at 590-630 nm for 1-2 minutes.	Record the slope (ΔA/min) for each well.

Plate Map :
Include columns for Blank (Buffer + DMSO), 100% Inhibition (Vehicle Control), 0% Inhibition (DMSO Control), and your compound concentrations.

Dissolve test inhibitors in proper solvent (e.g. DMSO). Dilute to 10X the desired test concentration with COX Assay Buffer before use. Add 10 µl diluted test inhibitor or Assay Buffer into assigned wells as sample screen [S] or Enzyme Control [EC] (no inhibitor) respectively. Add 2 µl of Celecoxib and 8 µl COX Assay Buffer into one of the wells as Inhibitor Control [IC].
Note: Solvents used to solubilize the inhibitors might affect the enzymatic activity. If solvent effect on enzymatic activity is a concern, prepare a solvent control well with the same final concentration of the solvent as in the inhibitor sample as solvent control.
2. Reaction Preparation: Dilute COX Cofactor 200 times by adding 2 µl of COX Cofactor to 398 µl of COX Assay Buffer just before use. Mix well. Prepare Arachidonic Acid solution by adding 5 µl of supplied Arachidonic Acid to 5 µl of NaOH just before use. Vortex briefly to mix. Dilute Arachidonic Acid/NaOH solution 10 times by adding 90 µl ddH2O, vortex briefly to mix. Make as much as needed.

For each well, prepare 80 µl of master mix as follows:
Reaction Master Mix

COX Assay Buffer	76 µl	
COX Probe	1 µl	
Diluted COX Cofactor	2 µl	
COX-2	1 µl	

Add 80 µl of Reaction Mix into each well. Use a multi-channel pipette to add 10 µl of diluted Arachidonic Acid/NaOH solution into each well to initiate the reactions at the same time.

Measurement: Measure fluorescence (Ex/Em = 535/587 nm) kinetically at 25°C for 5-10 min. Choose two points (T1 and T2) in the linear range of the plot and obtain the corresponding fluorescence values (RFU1 and RFU2).

4. Calculation: Calculate the slope for all samples, including Enzyme Control (EC), by dividing the net ΔRFU (RFU2 – RFU1) values by the time ΔT (T2 – T1). Calculate % Relative Inhibition as follows:
% Relative Inhibition = Slope of EC−Slope of SSlope of EC X100

5. Data Analysis

1. **Calculate Reaction Velocity:** For each well, calculate the rate of the reaction (V) as the change in absorbance per minute (ΔA/min) from the kinetic read.
2. **Calculate Percentage Inhibition:**
   - Average the velocities for the 0% inhibition control wells (V_max, enzyme activity with no inhibitor).
   - Average the velocities for the 100% inhibition control wells (V_min, non-enzymatic background).
   - For each test well: % Inhibition = [1 - ((V_sample - V_min) / (V_max - V_min))] * 100
3. **Plot and Calculate IC50:**
   - Plot % Inhibition (Y-axis) vs. Log10[Compound] (X-axis) using non-linear regression analysis.
   - Fit the data to a standard four-parameter logistic/sigmoidal dose-response curve (variable slope). Software like GraphPad Prism is ideal for this.
   - The IC50 is the concentration of the compound that gives 50% inhibition calculated from the curve fit.
4. **Determine Selectivity Index (SI):**
   - Calculate the COX-2/COX-1 selectivity index: SI = IC50 (COX-1) / IC50 (COX-2)
   - A high SI indicates COX-2 selectivity (like Celecoxib). A low SI (~1) indicates non-selectivity (like Indomethacin).

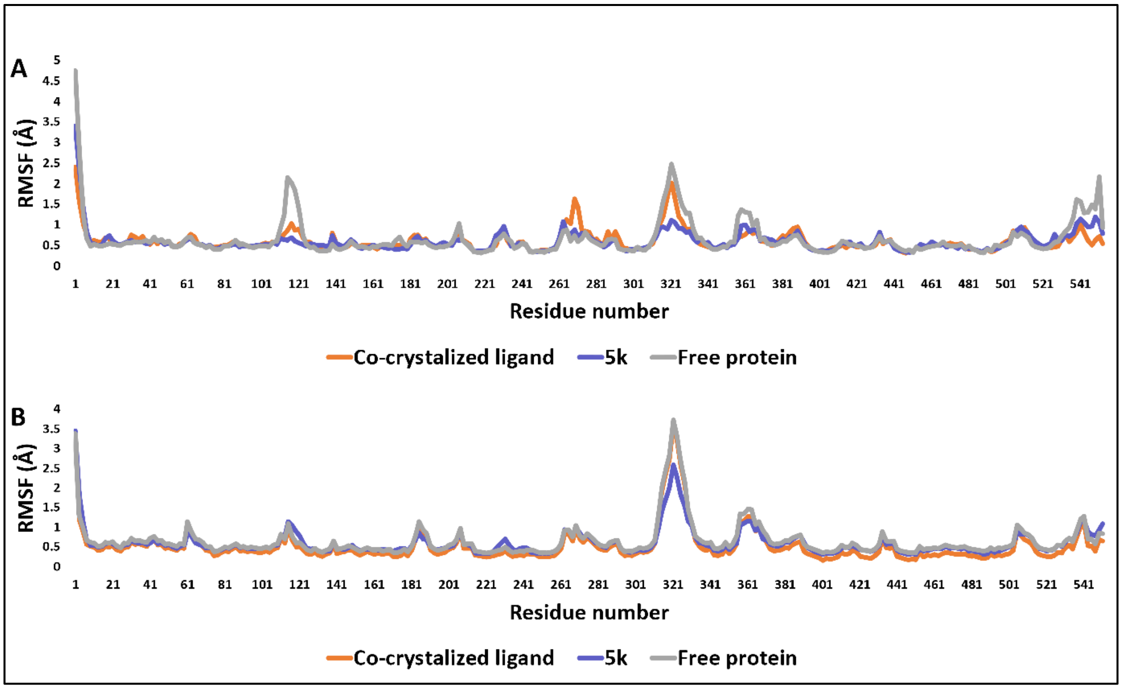

Figure 26. Root Mean Square Fluctuation (RMSF) plots of COX-1 (A) and COX-2 (B) in their apo form (gray), and in complex with compound **5k** (blue) and the co-crystallized ligand celecoxib (orange).

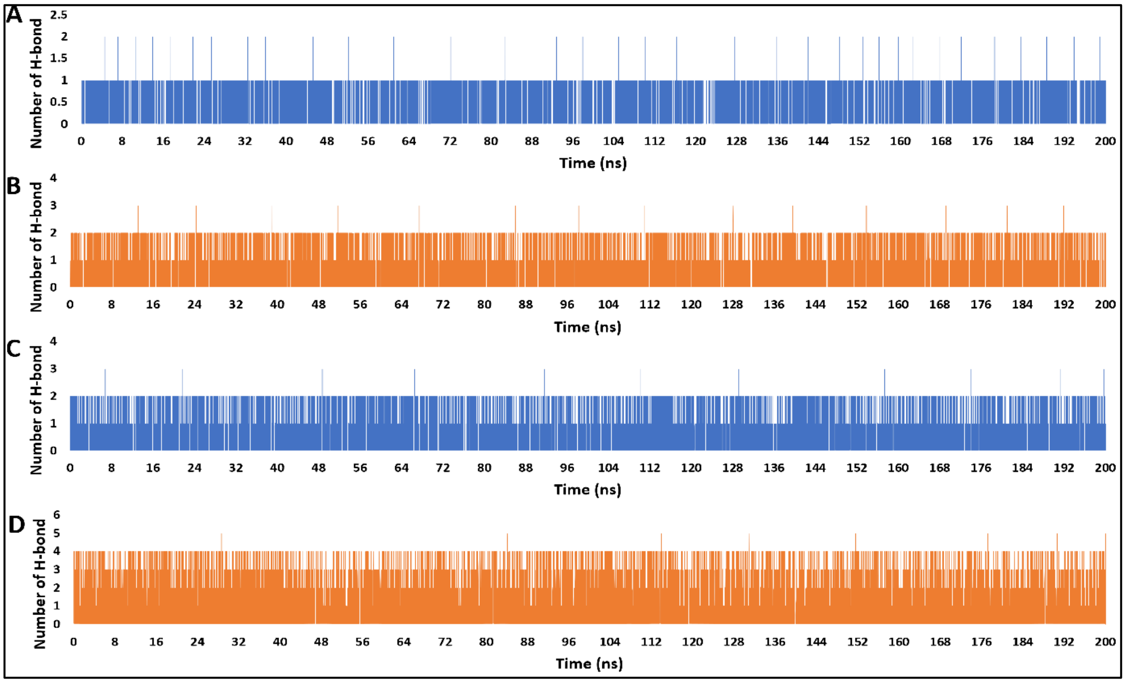

Figure 27. Hydrogen bond occupancy profiles for compound **5k** (A, C) and celecoxib (B, D) within the COX-1 (top) and COX-2 (bottom) active sites throughout 200 ns MD simulations.
